# Prostaglandin I_2_ signaling prevents angiotensin II-induced atrial remodeling and vulnerability to atrial fibrillation in mice

**DOI:** 10.1007/s00018-024-05259-3

**Published:** 2024-06-15

**Authors:** Yue Zhang, Meng Yuan, Wenbin Cai, Weiyan Sun, Xuelian Shi, Daiqi Liu, Wenhua Song, Yingqun Yan, Tienan Chen, Qiankun Bao, Bangying Zhang, Tong Liu, Yi Zhu, Xu Zhang, Guangping Li

**Affiliations:** 1 Tianjin Key Laboratory of Ionic-Molecular Function of Cardiovascular Disease, Department of Cardiology, Tianjin Institute of Cardiology, The Second Hospital of Tianjin Medical University, Pingjiang Road 23rd, Tianjin, 300211 China; 2 Tianjin Key Laboratory of Metabolic Diseases, Key Laboratory of Immune Microenvironment and Disease-Ministry of Education, Department of Physiology and Pathophysiology, Collaborative Innovation Center of Tianjin for Medical Epigenetics, Tianjin Medical University, Qixiang Tai Road 22nd, Tianjin, 300070 China; 3 Department of Cardiac Surgery, The Second Hospital of Tianjin Medical University, Pingjiang Road 23rd, Tianjin, 300211 China; 4 Department of Cardiology, First Affiliated Hospital of Kunming Medical University, Xichang Road 295th, Kunming, 650032 China; 5 Department of Physiology and Pathophysiology, Tianjin Medical University, Qixiang Tai Road 22nd, Tianjin, 300070 China; 6 Department of Cardiology, The Second Hospital of Tianjin Medical University, Pingjiang Road 23rd, Tianjin, 300211 China

**Keywords:** Prostaglandin I_2_, Atrial fibrillation, Atrial fibroblast, IL-6, IP receptor

## Abstract

Atrial fibrillation (AF) is the most common arrhythmia, and atrial fibrosis is a pathological hallmark of structural remodeling in AF. Prostaglandin I_2_ (PGI_2_) can prevent the process of fibrosis in various tissues via cell surface Prostaglandin I_2_ receptor (IP). However, the role of PGI_2_ in AF and atrial fibrosis remains unclear. The present study aimed to clarify the role of PGI_2_ in angiotensin II (Ang II)-induced AF and the underlying molecular mechanism. PGI_2_ content was decreased in both plasma and atrial tissue from patients with AF and mice treated with Ang II. Treatment with the PGI_2_ analog, iloprost, reduced Ang II-induced AF and atrial fibrosis. Iloprost prevented Ang II-induced atrial fibroblast collagen synthesis and differentiation. RNA-sequencing analysis revealed that iloprost significantly attenuated transcriptome changes in Ang II-treated atrial fibroblasts, especially mitogen-activated protein kinase (MAPK)-regulated genes. We demonstrated that iloprost elevated cAMP levels and then activated protein kinase A, resulting in a suppression of extracellular signal-regulated kinase1/2 and P38 activation, and ultimately inhibiting MAPK-dependent interleukin-6 transcription. In contrast, cardiac fibroblast-specific *IP*-knockdown mice had increased Ang II-induced AF inducibility and aggravated atrial fibrosis. Together, our study suggests that PGI_2_/IP system protects against atrial fibrosis and that PGI_2_ is a therapeutic target for treating AF.

The prospectively registered trial was approved by the Chinese Clinical Trial Registry. The trial registration number is ChiCTR2200056733. Data of registration was 2022/02/12.

**Supplementary Information:**

The online version contains supplementary material available at 10.1007/s00018-024-05259-3.

## Introduction

Atrial fibrillation (AF) is a highly prevalent cardiac arrhythmia that is associated with significant morbidity and mortality [1]. AF can increase the risk of stroke, heart failure, and other heart-related complications. Evidence suggests that atrial fibrosis is involved in the occurrence and maintenance of AF, and the degree of atrial fibrosis is a strong predictor of AF outcome [2]. Cardiac fibroblasts are important effector cells in the pathogenesis of cardiac fibrosis. Atrial fibroblasts, unlike their ventricular counterparts, are more responsive to mitogenic factors, such as angiotensin II (Ang II) [3]. Experimental animal models and human studies indicate that Ang II may be involved in the mechanism of atrial remodeling and AF [4–6].

Eicosanoids are hundreds of metabolites derived from polyunsaturated fatty acids, such as arachidonic acids, formed by the cyclooxygenase, lipoxygenase, and CYP450 pathways. Many eicosanoids play important roles in the cardiovascular system. The Framingham Heart Study found that six eicosanoids were significantly associated with incident AF after adjusting for clinical risk factors for AF [7]. The pro-inflammatory dihydroxyeicosatrienoic acids generated from arachidonic acid have been shown to induce atrial structural and electrical remodeling and increase atrial arrhythmia inducibility in the pressure-overload mice model [8]. In this study, we used targeted metabolomics to screen changes in the eicosanoid profile in patients with persistent AF (PAF) and investigated the potentially functional eicosanoids involved in AF.

Prostaglandin I_2_ (PGI_2_) is derived from the sequential metabolism of arachidonic acid by cyclooxygenase-2 (COX-2) and PGI synthase (PTGIS) upon stimulation by cytokines, growth factors, or other exogenous physical and chemical stimuli [9], which has a half-life of 2 minutes and quickly metabolizes into 6-keta prostaglandin-F1 alpha (6k-PGF1α). PGI_2_ is primarily synthesized in vascular endothelial and smooth muscle cells but is also synthesized in fibroblasts [10–12]. It inhibits platelet aggregation, smooth muscle contraction, and immune cell proliferation [13]. PGI_2_ has been recognized as a potent anti-fibrotic agent, which inhibits the synthesis of the extracellular matrix (ECM) [14] and cardiomyocyte hypertrophy [15]. The effects of PGI_2_ are mediated by the activation of the IP receptor, a Gs-type G protein-coupled receptor, which leads to an increase of intracellular cyclic adenosine 3’,5’-monophosphate (cAMP) and activation of protein kinase A (PKA). A series of studies have indicated that activation of intracellular cAMP signaling in cardiac fibroblasts promotes anti-fibrotic effects via inhibiting cell proliferation and decreasing ECM protein synthesis [16]. However, it is not yet known whether PGI_2_ exerts significant anti-fibrotic effects in the context of atrial fibrosis and AF.

Herein, we show that PGI_2_ content was significantly decreased in patients with AF. Furthermore, we find that PGI_2_ was mainly derived from atrial fibroblasts and a significant reduction in atrial tissues from patients and animal models with AF. We hypothesized that PGI_2_/IP receptor system prevents atrial remodeling and decreases vulnerability to AF. We indeed demonstrate that PGI_2_ inhibited atrial fibroblast differentiation, collagen synthesis, and interleukin-6 (IL-6) production in response to Ang II, partially through suppressing mitogen-activated protein kinase (MAPK) signaling activity. Our work reveals that PGI_2_ as an endogenous anti-fibrotic regulator may be a potentially therapeutic strategy for the treatment of AF.

## Materials and methods

### Reagents

Angiotensin II (Ang II) (ab120183) was from Abcam (Cambridge, UK). Iloprost (HY-A0096) was from MedChemExpress (Monmouth Junction, NJ, USA). Rp-8-Br-cAMPS (sc-3539) was from Santa Cruz Biotechnology (Dallas, TX, USA). Collagenase II (C8150) and rat tail tendon collagen (C8062) were from Solarbio (Beijing, China).

### Study design and overall protocol

Sinus rhythm (SR) controls and patients with AF were recruited from the Department of Cardiology, Second Hospital of Tianjin Medical University, starting from June 2020 to March 2021. The human left atrium (LA) tissues were obtained from patients undergoing coronary artery bypass graft surgery and venous blood samples were obtained after an overnight fast.

### Study population

The inclusion criteria for patients were as follows: (1) a clear clinical diagnosis of AF (electrocardiogram records showed that AF lasted more than 30 s and complied with the guidelines for radiofrequency ablation treatment indications); (2) first time receipt of AF catheter ablation; (3) age > 18 years, either sex; and (4) signed informed consent to voluntarily participated in the study. Exclusion criteria were as follows: (1) presence of severe heart disease, including New York Heart Association Functional Classification type 3 and 4, inability toile supine, and sever arrhythmia or hypertrophic cardiomyopathy; (2) life expectancy < 1 year; (3) past AF ablation history; (4) abnormal blood coagulation or anticoagulant drug allergy; (5) history of malignant tumor; (6) pregnancy; (7) uncontrolled thyroid disease; (8) severe electrolyte disturbance; (9) polyarteritis; (10) acute inflammation; and (11) severe liver and kidney dysfunction (glutamate or aspartate aminotransferase > 120 U/L, or serum creatinine > 132 µmol/L).

### Metabolomics profiling

Metabolomic analysis involved liquid chromatography tandem mass spectrometry (LC-MS/MS) of metabolites as we previously described [17–19]. Briefly, 200 µL plasma samples and 50–100 mg atrial tissues were spiked with internal standard mixture (5 ng) and extracted by solid-phase extraction, then cartridges were washed with 2 mL of 5% methanol and pumped vacuum. Analytes were eluted with methanol and evaporated to dryness. The residues were dissolved in 100 µL of 30% acetonitrile. Chromatography separation involved use of an UPLC BEH C18 column (1.7 μm, 50 × 2.1 mm i.d.) consisting of ethylene-bridged hybrid particles (Waters, Milford, MA). Target profiling of polyunsaturated fatty acid involved use of the 5500 QTRAP hybrid triple quadruple linear ion-trap mass spectrometer (AB Sciex; Foster City, CA, USA) equipped with a turbo ion-spray electrospray ionization source.

### Animal studies

Male C57BL/6J mice (6–8 weeks of age) were selected to be surgically implanted with ALZET osmotic mini-pumps (Model 1004; Durect Corporation, Cupertino, CA, UAS) to infuse saline or Ang II (2000 ng/kg per min) for 28 days as previously described [4]. All mice were maintained under a 12:12 h light/dark cycle (lights on at 7:00 and lights off at 19:00) before and throughout experiments.

### Administration of synthetic PGI_2_ analog iloprost

The iloprost power, a synthetic PGI_2_ analog, behaved as a full agonist of IP receptor and was dissolves in 10% dimethyl sulfoxide and 90% corn oil. Starting from the third week after Ang II infusion, mice were administered iloprost (0.2 mg/kg per day) [20] or vehicle via intraperitoneally injection for a duration of two weeks. Atrial tissues were removed for further analysis.

### Blood pressure (BP) measurements

BP was monitored by a tail-cuff system (BP-98AL; Softron, Tokyo, JP). Mice were trained for 1 week to be acquainted with the measurement. BP values were averaged from at least three consecutive measurements for each mouse. BP was determined for 3 consecutive days starting from 3 days before and at day 7, day 14, day 21 and day 28 after Ang II treatment.

#### Delivery of adeno-associated virus

Adeno-associated virus serotype 9 (AAV9), containing a periostin promoter driving the expression of shRNA targeting IP, was obtained from Genechem (Shanghai, China) as previously described [21]. This AAV construct was systemically injected to specifically target gene expression in myofibroblast-like lineage cells within the hearts of mice. Wild-type mice (C57BL/6 background) received injections of AAV9 carrying the periostin promoter-shIP construct, resulting in knockdown of the IP receptor in cardiac fibroblasts, referred to as AAV-POSTN-shIP, or AAV9 carrying periostin promoter-scramble construct as a control, via tail-vein administration (150 µL; 1.5 × 10^11^ v.g). Following a 2-week post-injection period, mice were administered Ang II at a dose of 2000 ng/kg per minute or saline for an additional 28 days. Atrial tissues were then collected for further analysis.

### Echocardiographic measurements of the LA

Echocardiography was conducted following established procedures [22]. Briefly, mice were anesthetized with 1% isoflurane at a flow rate of 1 L/min and placed in a supine position on the platform. Using a Vevo2100 system equipped with MS400 transducer (FUJIFILM VisualSonics, Toronto, CA, USA), a parasternal long-axis view was obtained to visualize the LA within the 2D image. The inner edge of the LA was manually traced at end-systole using the Vevo software’s measurement tools to determine its diameter. This process repeated over multiple cardiac cycles for accuracy, the average left atrial diameter (LAD) was computed for analysis. Additionally, a parasternal short-axis view of the left ventricle was obtained at the level of the papillary muscles using either M-mode or B-mode to visualize the ventricle. The left ventricular internal dimension in diastole (LVID; d) was measured at end-diastole, and the left ventricular internal dimension in systole (LVID; s) was measured at end-systolic. These measurements were utilized to calculate the left ventricular ejection fraction (LVEF) using the formular: LVEF=[(LVID; d-LVID; s)/LVID; d]×100%. The parameters LAD and LVEF were calculated using proprietary software provided by the Vevo system (VisualSonics Vevo Lab software, version 1.6.0). Each measurement was performed three times, and the average value was used for analysis by two experienced readers.

### Induction of AF

AF conduction in mice was performed as previously described [23, 24]. In brief, an 8-electrode catheter (1.1 F octapolar electrophysiology catheter, EPR-800; Millar Instruments, Houston, TX, USA) was inserted via the jugular vein into the right atrium and ventricular. Atrial arrhythmias inducibility was assessed using an automated simulator connected to the data acquisition system (3008-FA; Multi Channel Systems, Reutlingen, German). This involved delivering eleven sets of 2-second tachypacing bursts through the catheter electrodes, starting with an initial burst at a cycle length of 40 ms and subsequent bursts decreasing by 2 ms increments until reaching a final cycle length of 20 ms. This sequence of burst stimulation was repeated twice. AF was defined as a rapid and irregular atrial rhythm with irregular RR intervals lasting for 1 s or more on surface electrocardiogram (ECG). Successful AF induction required 2 or more bursts out of the 3 series administered. AF episodes were identified from direct atrial activation recordings using LabChart8 software (ADInstruments, New South Wales, AUS).

### Epicardial electrical activation mapping

LA electrograms were captured utilizing a 6 × 6 multiple-electrode probe array (MEPAs), where recording electrodes were pressed against the epicardial surface of the anterior aspect of the LA free wall. The interelectrode distance was 0.4 mm, resulting in a recording area of 2.0 × 2.0 mm. Data acquisition was facilitated by a multi-electrical array mapping system (EMS64-USB-1003; MappingLab., Oxford, UK), with activation waveforms amplified via a filter amplifier and transmitted to a connected computer. Activation times at each electrode were determined as the time at the point of maximum negative deflection relative to the earliest fiducial point of activation. Color-coded activation maps were generated using MATLAB. Conduction vectors were used to calculate the average conduction velocity at the recording site. The phase, expressed in ms/mm for each electrode, was calculated based on the difference in activation time between an electrode and its neighboring electrodes divided by the inter-electrode distance [25]. Phase differences in activation, absolute inhomogeneity (the range of phase differences from the 5th-95th percentiles), and the index of inhomogeneity (the absolute inhomogeneity divided by the median of the phase differences) were computed according to the method of Lammers et al. [26].

### Histology

The atrial tissue was excised from human and mice and the process of paraformaldehyde-fixing and paraffin-embedding. Section (5-µm-thick) of atrial tissue were deparaffinized and stained with Sirus red. Image-Pro 7.0 (Maryland, USA) analyses were used to quantitative atrial fibrosis.

### Immunofluorescence staining

Sections measuring 4 μm were cut off from paraffin-embedded tissue block. Immunofluorescence staining was performed with antibodies against Vimentin (5741 S; Cell Signaling Technology, Danvers, MA, USA); α-actinin (ab68194), CD31 (ab76533), α-SMA (ab7817, ab124964) and Ki67 (ab15580) (all Abcam, Cambridge, UK); PTGIS (sc-293247), IL-6 (sc-28343) (all Santa cruz Biotechnology, Dallas, TX, USA); Alexa Fluor 594-conjugated wheat germ agglutinin (W11262; Thermo Fisher Scientific, Waltham, MA, USA), according to the manufacture’s instruction. Confocal immunofluorescence images were captured by confocal laser scanning microscopy (FV1000-IX81; Olympus, Tokyo, Japan).

### Isolation and culture of mice atrial fibroblasts

The method of isolating primary mice atrial fibroblasts was performed as previously described [27]. Briefly, atrial tissues were harvested by gentle trituration and centrifuged at 800 rpm for 5 min to separate cardiomyocytes from fibroblasts. The supernatant was collected and centrifuged at 2000 rpm for 10 min to pellet fibroblasts. Atrial fibroblasts were collected in DMEM (Dulbecco’s Modified Eagle Medium)-F12 supplemented with 20% fetal bovine serum and 1% penicillin/streptomycin. Cells were plated and cultured for 5–7 days to reach confluence and maintained in 5% CO_2_/95%-humidified air at 37 °C. Fibroblasts at passages 3 to 5 were used in experiments.

### cAMP (cyclic adenosine monophosphate) assay

Primary atrial fibroblasts were seeded at 25,000 cells/well into 96-well plates, and grown overnight in complete medium. Next, removed media and replaced with serum-free media containing 1 mM 3-isobutyl-1-methylxanthine (Sigma-Aldrich; Burlington, MA, USA) for 20 min, then subjected to experimental treatment. Cells lysed after appropriate response time and cAMP levels were determined using the cAMP-Screen® System (4412183; Thermo Fisher Scientific, Waltham, MA, USA). Luminescence was read with Cytation 3 M plate reader (Bio Tek Ins., Winooski, VT, USA).

### PKA assay

ELISA-based assay kit (ab139435; Abcam, Cambridge, UK) was used to measure PKA activity in cell lysates as previously described [28]. Cells were lysed in Lysis Buffer for 10 min, centrifuged at 13,000 rpm for 15 min to remove insoluble material and protein was quantified using BCA method (23225; Thermo Fisher Scientific, Waltham, MA, USA). Lysates were diluted in the kit dilution buffer as required to load 2,000 ng of total cellular protein in 30 µL per well of the assay plate. Initiate reaction by adding 10 µL of reconstituted ATP and 40 µL PKA phosphospecific substrate antibody, respectively. Measure absorbance increase on Cytation 3 M microplate reader (Bio Tek Ins., Winooski, VT, USA).

### RNAi

Atrial fibroblasts were transfected with two independent siRNA directed against mouse IP (SASI_Mm01_00138200/SASI_Mm02_00315041; Sigma-Aldrich; Burlington, MA, USA), or respective negative control siRNA (MISSION® siRNA Universal Negative Control, Cat No. SIC001/SIC002; Sigma-Aldrich; Burlington, MA, USA). Transfection complexes were prepared with Lipofectamine 3000 (L3000001; Thermo Fisher Scientific, Waltham, MA, USA) according to the manual. For each well of a 6-well plate, 75 pmol of siRNA was diluted in Opti-MEM (31985062; Thermo Fisher Scientific, Waltham, MA, USA) giving a final volume of 250 µL. After incubating the mixture for 15 min at ambient temperature, the solution was added onto the cells, which had been pre-incubated tin 1 mL Opti-MEM prior to the transfection. Cells were then incubated at 37 °C and 5% CO_2_. After 6 h, cells were washed, and normal cell medium was added to the cells followed by incubation for 48 h for knockdown of IP.

### Total RNA extraction and real-time PCR analysis

Total RNA was extracted from cells by using RNA extraction kits (LS1040; Promega, Madison, WI, USA). RNA samples were reverse-transcript with SuperScript and random primers (A2800; Promega, Madison, WI, USA). Real-time PCR involved use of the SYBR Green qPCR Master Mix (A6001; Promega, Madison, WI, USA) and the ABI 7500 Real-Time PCR System (Life Technologies, Carlsbad, CA, USA). Gene expression was normalized to *Actb* level. Primer sequences are in Table S1.

### Western blot analysis

Total protein were extracted from cells by use of RIPA buffer (R0010; Solarbio, Beijing, China) containing Complete Protease Inhibitor Cocktail Tablets and phosphorylase inhibitor (11697498001, 04906845001; Roche, Basel, Switzerland). Protein extracts were subjected to SDS-PAGE, transferred to polyvinylidene fluoride membranes (A29562259; GE Healthcare Life Sciences, Chicago, Illinois, USA). Analysis involved the primary antibodies for p-P38 (9211 S), P38 (8690 S), p-JNK (9255 S), JNK (9252 S) and IL-6 (12912S) (all Cell Signaling Technology, Danvers, MA, USA); p-ERK (ab50011), ERK (ab17942), α-SMA (ab7187), Collagen I (ab34710) and Periostin (ab14041) (all Abcam, Cambridge, UK); PTGIS (sc-293247) and IP (sc-365268) (all Santa cruz Biotechnology; Dallas, TX, USA).

### Cell viability assay

Atrial fibroblasts were plated in 96-well plates (1,000 cells per well, triplicate) in 100 µL conditioned medium. After experimental treatment, cells were replaced with 90 µL fresh growth medium supplemented with 10 µL MTS reagents (ab197010; Abcam, Cambridge, UK) followed by incubation at 37 °C for 1 h. The absorbance optical density value was measured at 490 nm using a Cytation 3 M plate reader (Bio Tek Ins., Winooski, VT, USA).

#### Cell contraction assay

The assay was performed with Collagen-based Contraction Assay Kit (CBA-201; Cell Biolabs, San Diego, CA, USA) according to the supplied manual. Atrial fibroblasts were embedded in collagen gel (2 × 10^6^ cells/mL gel) in a 48-well pate format (250 µL gel/well converted by 500 µL complete medium). The next day, cells were starved for 24 h, gel lattices released and then experimentally treated for additional 24 h. Images were acquired immediately after gel release and then after 24 h of treatment on BX53 (Olympus, Tokyo, Japan). The quantifications of gel size were performed in Image-Pro 7.0 (Maryland, USA).

### Cell migration assay

Migration of atrial fibroblasts was measured by wound-healing assay. Fibroblasts were grown to confluence in 6-well plates and the bottom monolayer of cells was scraped away using a sterile 200 µL pipette tip. After experimental treatment, for each well, images of four to five randomly selected regions were captured at 0 and 24 h under an Olympus inverted microscope. The relative speed of fibroblasts migration was calculated as the mean linear movement of fibroblasts over wound edges at 24 h. Further, migrated fibroblasts over time normalized with the migration of untreated cells and expressed as a fold change from controls.

### ELISA

Atrial fibroblasts were seeded at a density of 20,000 cells/well onto fibronectin-coated 96-well plates in 100 µL/well complete growth medium. After experimental treatment, the concentrations of IL-6 in cell supernatant and mice plasma were determined by Mouse IL-6 ELISA Kit (ab222503; Abcam, Cambridge, UK) according to manufacturer’s manual. The absorbance was recorded on Cytation 3 M microplate reader (Bio Tek Ins., Winooski, VT, USA).

### Statistical analysis

Data are presented as mean ± SEM. SPSS Statistics v23.0 (IBM Corp, Armonk, NY, USA) and GraphPad Prism v8.0 (GraphPad Software, San Diego, CA, USA) were used for all statistical analyses and graph production. An unpaired Student *t*-test (two-tailed), one-way ANOVA, or two-way ANOVA with the Bonferroni multiple comparison post hoc test was used for analysis. Statistical significance was set at *P* < 0.05.

Analyst v1.5.1 software (SCIEX, Framingham, MA, USA) was used to process the raw metabolite profile data. MetaboAnalyst v5.0 (http://www.metaboanalyst.ca) was used to perform partial least squares discriminant analysis (PLS-DA) [29]. Data were log-transformed and auto-scaled before analysis. Global changes between samples from the participant groups were compared using non-parametric tests with a fold-change threshold of two.

## Results

### PGI_2_ content was decreased in patients with AF

**Fig. 1:**
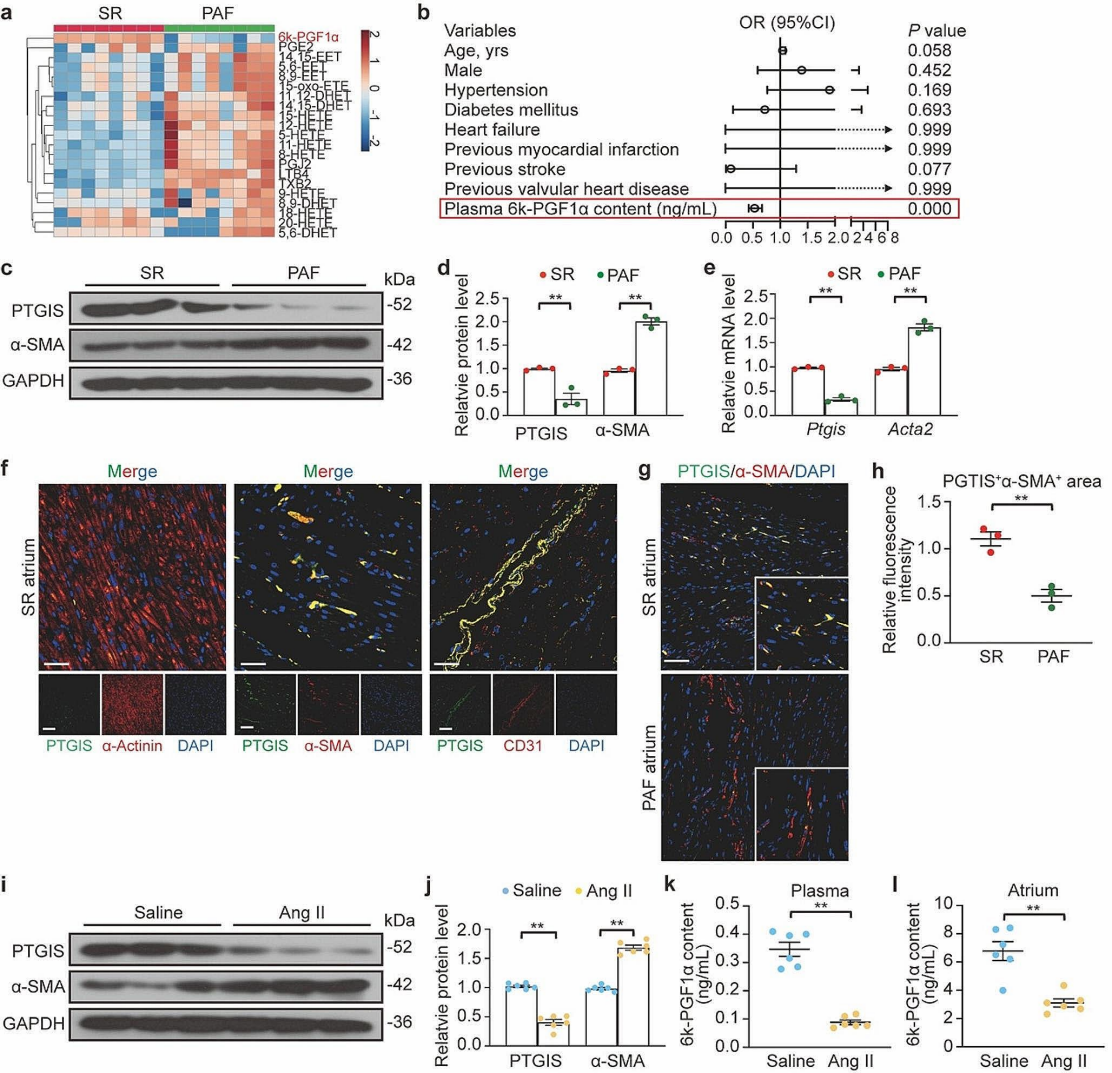
PGI_2_(prostaglandin I_2_) production was decreased in patients with AF (atrial fibrillation) and Ang II (angiotensin II)-infused mice. PGI_2_ was determined as its derivative 6k-PGF1α. **a**, LC-MS/MS (liquid chromatography-tandem mass spectrometry) detection of arachidonic acid-derived metabolites in the plasma of SR (sinus rhythm) controls and patients with AF. Heat map showing eicosanoid profiles of arachidonic acid in SR (*n* = 8) and PAF (persistent AF, *n* = 8) groups. Clustering algorithm model is single. **b**, Forest plot representation for 6k-PGF1α content and clinical factors associated with AF occurrence according to logistic regression analysis. OR: odds ratio; CI: confidence interval. (SR, *n* = 55; AF, *n* = 182) **c** and **d**, Western blot analysis (**c**) and quantification (**d**) of PTGIS and α-SMA (alpha-smooth muscle actin) protein levels in the left atrium obtained from SR controls (*n* = 3) and patients with PAF (*n* = 3). **e**, Quantification of *Ptgis* (prostaglandin I synthase) and *Acta2* (actin alpha 2) mRNA levels in the left atrium from SR controls (*n* = 3) and patients with PAF (*n* = 3). **f**, Immunofluorescence staining of PTGIS in the left atrium of SR controls. Scale bare, 50 μm. **g** and **h**, Immunofluorescence staining (**g**) of PTGIS and α-SMA in the left atrium of SR controls (*n* = 3) and patients with PAF (*n* = 3) and quantification (**h**) of relative fluorescence intensities of colocalization. Scale bare, 50 μm. **i** though **l**, C57BL/6J mice were infused with saline (*n* = 6) or Ang II (2000 ng/kg/min, *n* = 6) for 28 days. **i** and **j**, Western blot analysis (**i**) and quantification (**j**) of PTGIS and α-SMA protein levels in mouse atrial tissues at 28 d. **k** and **l**, LC-MS/MS detection of plasma (**k**) and atrial tissue (**l**) levels of 6k-PGF1α in mice at 28 d. Unpaired 2-tailed *t*-test, ^**^P < 0.01

Several studies have indicated that prostaglandins play an important role in tissue fibrosis [30]. To elucidate the relationship between AF and prostaglandin production, we utilized a cohort of human plasma samples collected from patients with persistent atrial fibrillation (PAF) and those with normal SR (Table 1). The levels of 21 detectable arachidonic acid-derived oxylipins were measured using LC-MS/MS and shown as a heatmap (Fig. 1a). A PLS-DA score plot showed that the classification model could separate the samples according to their group (Fig. S1A). Table 2 presents the levels and fold changes of each significantly different eicosanoid between the patients with PAF and SR controls. Notably, the PGI_2_ content, determined by its derivative 6k-PGF1α, showed a significant decrease in the PAF group (Fig. S1C). Additionally, the levels of eight other eicosanoids were significantly increased in the same group. The variable important for prediction scores indicated that PGI_2_ contributed more to the classification of the SR controls and patients with PAF (Fig. S1B). To further validate the role of PGI_2_ in the development of AF, we expanded our study cohort by enrolling additional patients, comprising 182 individuals with AF and 55 without AF. Through multiple logistic regression analysis, we found that lower plasma PGI_2_ levels (0.99 ± 0.09 ng/mL for SR controls versus 5.72 ± 0.88 ng/mL for patients with AF, OR = 0.531, 95%CI: 0.424–0.665, *P* = 0.000000034967) were significantly associated with an increased risk of AF (Table 3; Fig. 1b, Fig. S1D).

**Table 1:** Clinical characteristics of healthy controls and persistent AF patients

Variables	SR group (*n* = 8)	PAF group (*n* = 8)	*P* value^a^
Age (yr, range[median])	52–65 (58.5)	52–68 (61.6)	n.s.
Male (no. [%])	4/8 (50.0%)	5/8 (62.5%)	n.s.
HR (beats/min, range[median])	55–97 (72.8)	52–92 (73.8)	n.s.
SBP (mmHg, range[median])	109–158 (125.6)	109–150 (126.9)	n.s.
DBP (mmHg, range[median])	69–96 (79.6)	70–105 (79.9)	n.s.
Clinical features (no. [%])			
Current smoking	3/8 (37.5%)	2/8 (25.0%)	n.s.
Hypertension	5/8(62.5%)	7/8 (75.0%)	n.s.
Diabetes mellitus	0	0	n.s.
Previous myocardial infarction	0	0	n.s.
Previous stroke	0	1 (12.5%)	n.s.
Medication history (no. [%])			
Warfarin	0	0	n.s.
Aspirin	0	1/8 (12.5%)	n.s.
Beta-blocker	1/8 (12.5%)	2/8 (25)	n.s.
ACEI and/or ARB	3/8 (37.5%)	1/8 (12.5%)	n.s.
Statin	1/8 (12.5%)	0	n.s.
CCB	2/8 (25.0%)	3/8 (37.5%)	n.s.
Laboratory assessment (range[median])			
WBC (10^9^/L)	3.8–7.8 (6.3)	3.1–11.3 (6.8)	n.s.
Hb (g/L)	123–161 (137.4)	124–168 (148.0)	n.s.
Cr (µmol/L)	44.9–73.8 (57.4)	47.9–88.7 (62.2)	n.s.
UA (mmol/L)	185.5-423.8 (299.6)	242.4-541.8 (338.9)	n.s.
Na^+^ (mmol/L)	138.2-148.1 (142.9)	135.2–148.0 (142.8)	n.s.
K^+^ (mmol/L)	3.9–4.6 (4.1)	3.9–4.9 (4.4)	n.s.
Echocardiogram features (range[median])			n.s.
LAD (mm)	28.1–43.7 (35.7)	32.7–60.0 (42.5)	n.s.
LVEDD (mm)	45.2–50.4 (48.1)	44.8–54.3 (48.0)	n.s.
IVSd (mm)	7.5–8.5 (8.0)	6.8–11.4 (8.6)	n.s.
LVPWd (mm)	8.2–10.4 (9.3)	7.4–11.6 (9.1)	n.s.
EF (%)	55–68 (63.8)	55–71 (63.4)	n.s.

SR: sinus rhythm; PAF: persistent atrial fibrillation; HR: heart rate; SBP: systolic blood pressure; DBP: diastolic blood pressure; ACEI: angiotensin conversion enzyme inhibitor; ARB: angiotensin II receptor blocker; CCB: calcium channel blocker; WBC: white blood cells; Hb: hemoglobin; Cr: creatinine; UA: uric acid; LAD: left atrium dimension; LVEDD: left ventricular end-diastolic dimension; IVSd: diastolic interventricular septum diameter; LVPWd: diastolic left ventricular posterior wall diameter; EF: ejection fraction

Values are presented as mean ± SEM, or absolute numbers (%). n.s.: not significant

^a^Differences were evaluated using independent-samples *t* test or Chi-square test for SR group vs. PAF group

**Table 2:** Eicosanoids with significant change between normal controls and persistent AF patients

Eicosanoids (ng/mL, range[median])	SR group (*n* = 8)	PAF group (*n* = 8)	log2 (Fold change)	-log10 (*P* value)^a^
6k-PGF1α	9.59–26.92 (16.35)	0-2.95 (0.80)	4.34	3.03
PGJ_2_	0.02–0.05 (0.04)	0.07–0.17 (0.13)	-1.82	3.81
LTB4	0.05–0.13 (0.11)	0.41–1.32 (0.95)	-3.11	3.81
8-HETE	0.09–0.18 (0.13)	0.21–2.75 (1.07)	-3.06	3.81
11-HETE	0.10–0.25 (0.18)	0.35–5.51 (1.62)	-3.21	3.81
5-HETE	0.14–0.33 (0.25)	0.37–3.93 (1.43)	-2.54	3.81
15-HETE	0.44-1 (0.68)	1.21–7.25 (2.49)	-1.88	3.81
12-HETE	0.24–0.79 (0.53)	0.71–13.47 (3.42)	-2.69	3.21
15-oxo-ETE	0-0.54 (0.11)	0.1–1.77 (0.71)	-2.73	2.33

SR: sinus rhythm; PAF: persistent atrial fibrillation

^a^Significant change in level indicated fold change > 2 or < 50%, *P* value versus SR group

**Table 3:** Clinical characteristics of healthy controls and AF patients

Variables	SR group (*n* = 55)	AF group (*n* = 182)	*P* value^a^
Age (yr, range[median])	39–83 (65.1)	41–87 (67.4)	n.s.
Male (no. [%])	25/55 (45.6%)	94/182(51.7%)	n.s.
Hypertension	35/55 (63.6%)	131/182 (80.0%)	n.s.
Diabetes mellitus	4/55 (7.3%)	10/182 (5.5%)	n.s.
Heart failure	0	5/182 (2.8%)	n.s.
Previous myocardial infarction	0	13/182 (7.1%)	n.s.
Previous stroke	3/55 (5.5%)	3/182 (1.7%)	n.s.
Previous valvular heart disease	0	4/182 (2.2%)	n.s.
Plasma 6k-PGF1α content (ng/mL, range[median])	0.17–31.63 (5.72)	0-8.48 (0.99)	0.000

SR: sinus rhythm; AF: atrial fibrillation

n.s.: not significant

^a^Differences were evaluated using independent-samples *t* test or Chi-square test for SR group vs. AF group

To elucidate why patients with PAF exhibited reduced PGI_2_ production, we analyzed PTGIS in the LA tissue of such patients. The expression of PTGIS was lower in the PAF group compared with those in SR controls at both mRNA level and protein level (Fig. 1c to e), accompanied by elevated alpha-smooth muscle actin (α-SMA) levels, and subepicardial and myocardial fibrosis (Fig. S1E and S1F). Furthermore, we found that PTGIS colocalized with the fibroblast marker α-SMA and the endothelial cell marker CD31, but not with the cardiomyocyte marker α-actinin (Fig. 1f). Double fluorescence staining demonstrated a marked decrease in PTGIS in fibroblasts of the LA tissue from patients with PAF, paralleling the upregulation of α-SMA (Fig. 1g and h). Taken together, these observations demonstrate that PTGIS is specifically downregulated in fibroblasts in the atrial tissue of patients with AF. These results were confirmed using the Ang II-induced AF mouse model. PTGIS levels were downregulated in Ang II-treated atrial tissues compared to those treated with saline control (Fig. 1i and j). Moreover, PGI_2_ content was reduced in the presence of both plasma and atrial tissue from the Ang II-infused mice (Fig. 1k and l). These results imply that PGI_2_ participates in the development and progression of AF.

### Treatment with the PGI_2_ analog iloprost reduced Ang II-induced AF

**Fig. 2:**
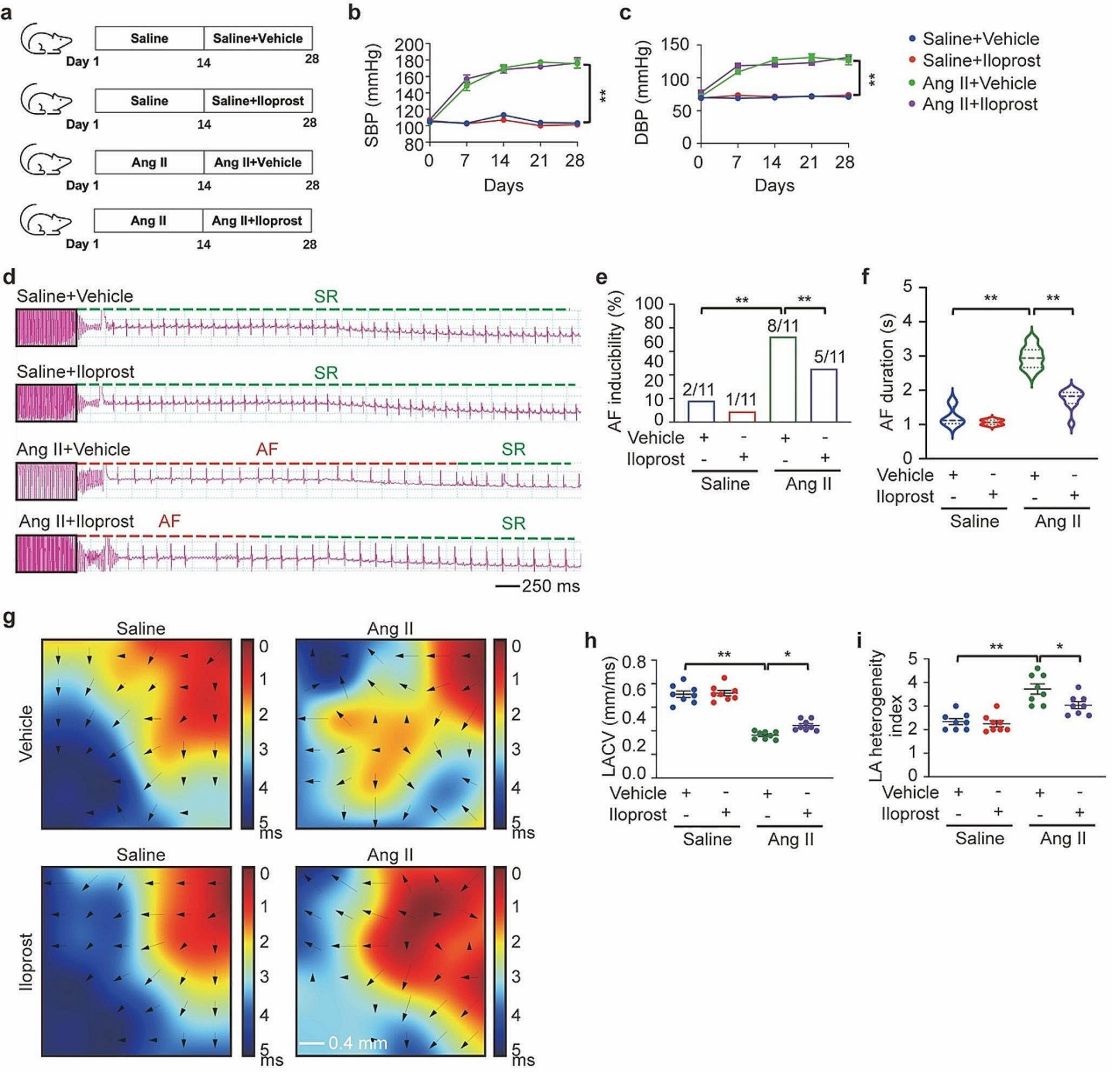
PGI_2_(prostaglandin I_2_) analog iloprost reduced Ang II (angiotensin II)-induced AF (atrial fibrillation) inducibility. **a**, C57BL/6J mice were housed with or without infusion of Ang II (2000 ng/kg/min) for 28 days. Starting on the 15th day of infusion, iloprost (0.2 mg/kg/day) or vehicle were supplied for a period of 14 days. **b** and **c**, Noninvasive tailed-cuff monitoring of SBP (systolic blood pressure, **b**) and DBP (diastolic blood pressure, **c**) of Saline/Vehicle, Saline/Iloprost, Ang II/Vehicle, and Ang II/Iloprost mice. **d**, Representative surface ECG (electrocardiogram) tracing during burst pacing (BP) is depicted within the black box. Red dashes denote AF, and green dashes denote sinus rhythm (SR). **e**, Fraction of animals with successful AF induction. Ratios represent the number of AF induced to total animals. *n* = 11 per group, Fisher exact test. **f**, Violin plot of AF in animals (numbers for each group are shown in **e**). **g**, Representative epicardial electrical conduction activation maps of the left atrium. The color bar on the right side of the image represents the conduction proceeding advancing from 0 ms (dark red) to the end of imaging period (5 ms, blue). The arrows indicate the direction of conduction. Scale bare, 0.4 mm. **h**, Summary of CV (conduction velocity) in the left atrium. **i**, Summary of inhomogeneity index in the left atrium. Two-way ANOVA, *n* = 8–11 mice per group, ^*^P < 0.05, ^**^P < 0.01

To investigate whether PGI_2_ regulates AF development, mice infused with Ang II were treated with the PGI_2_ analog iloprost for two weeks (Fig. 2a). Iloprost is a synthetic PGI_2_ analog indicated to treat pulmonary arterial hypertension. Iloprost has greater chemical stability than PGI_2_, which facilitates its clinical use. Systolic blood pressure (SBP) and diastolic blood pressure (DBP) were elevated in Ang II-treated mice, with or without iloprost treatment, but there was no statistically significant difference between the two groups (Fig. 2b and c). The inducibility of AF was increased in Ang II-treated mice (8/11) compared with saline-treated mice (2/11), and this effect was reduced in mice co-treated with Ang II and iloprost (5/11) (Fig. 2d and e). The duration of AF was shortened substantially in mice administered iloprost compared with vehicle-treated mice following Ang II infusion (Ang II + vehicle: 2.962 ± 0.081 s versus Ang II + iloprost: 1.747 ± 0.092 s) (Fig. 2f). There was no significant difference in the inducibility of AF between the saline groups, with or without iloprost (2/11 for saline + vehicle versus 1/11 for saline + iloprost) (Fig. 2e and f). Next, we recorded the electrical conduction mapping of the LA in vivo. LA electrical conduction in saline-infused mice, with or without iloprost, was uniform and showed an orderly spread to the surrounding tissue (Fig. 2g). However, LA electrical conduction in Ang II-infused mice was disordered, and there were abnormal positions in the wave conduction, which was restored after iloprost treatment (Fig. 2g). The left atrial conduction velocity was significantly lower in Ang II-infused mice and improved in the presence of iloprost (Fig. 2h). Moreover, LA conduction dispersion was increased in Ang II-infused mice and reduced in iloprost-treated mice (Fig. 2i).

## Administration of the PGI_2_ analog iloprost inhibited Ang II-induced atrial fibrosis

**Fig. 3:**
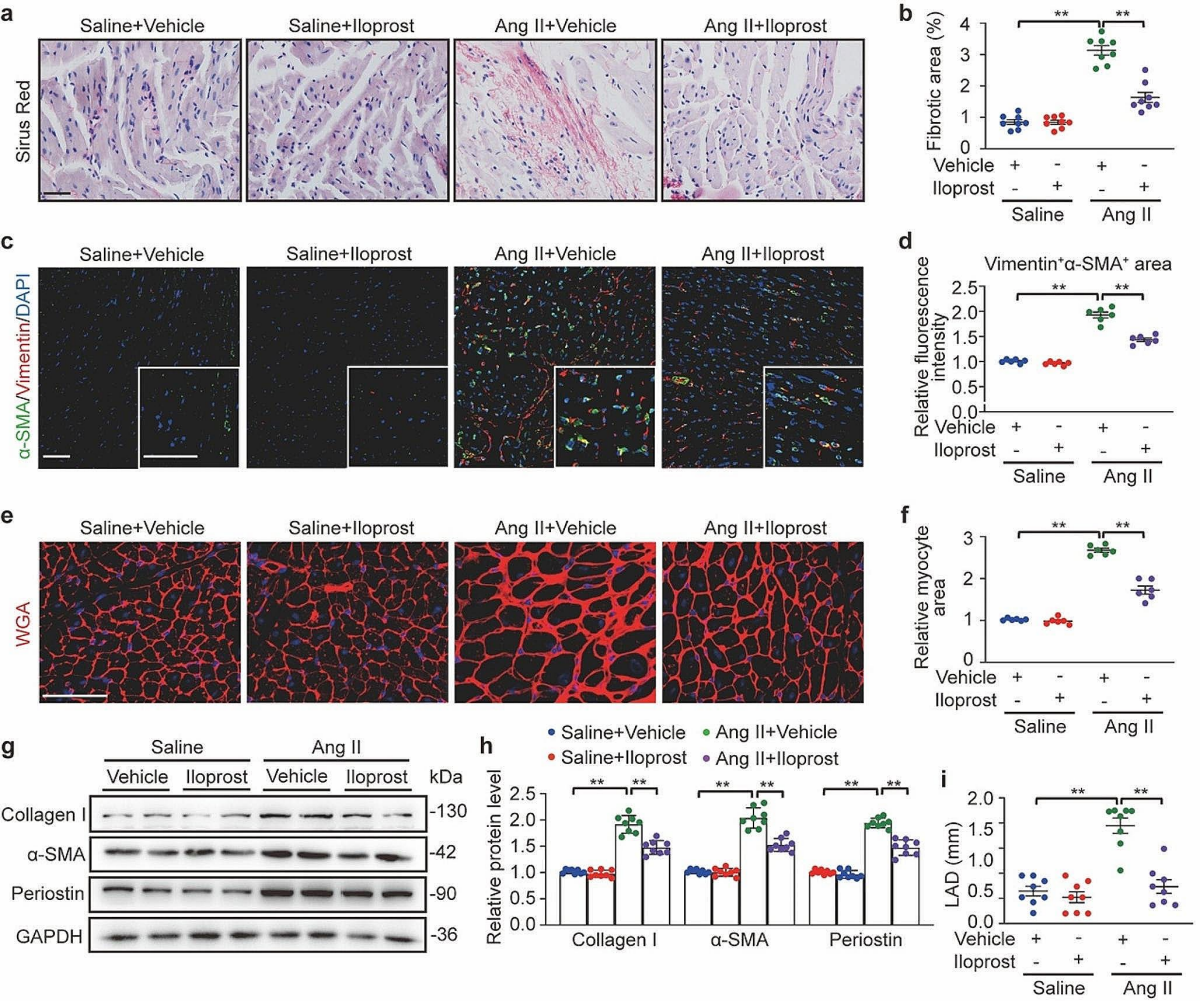
PGI_2_(prostaglandin I_2_) analog iloprost reduced Ang II (angiotensin II)-induced atrial fibrosis. **a**, Representative Sirus red staining of left atrial tissues of Saline/Vehicle, Saline/Iloprost, Ang II/Vehicle, and Ang II/Iloprost mice. Scale bare, 50 μm. **b**, Quantification of the fibrotic area. **c**, Representative immunofluorescence staining of α-SMA (alpha-smooth muscle actin) and Vimentin. in left atrial tissues in each group. Scale bare, 50 μm. **d**, Quantification of relative fluorescence intensities of colocalization. **e**, Alexa Fluor 594-conjugated wheat germ agglutinin (WGA) staining of left atrial tissues in each group. Scale bare, 50 μm. **f**, Quantification of the myocyte area. **g** and **h**, Western blot analysis (**g**) and quantification (**h**) of Collagen I, α-SMA and Periostin protein levels in the atrial tissues. **i**, Quantification of LAD (left atrium diameter) in each group. Two-way ANOVA, *n* = 6–8 mice per group, ^*^P < 0.05, ^**^P < 0.01

The effect of iloprost on atrial remodeling was investigated. Histological analysis of LA tissues from Ang II/iloprost-treated mice showed reduced disorganized myofibers and collagen deposition, as detected by Sirus Red (Fig. 3a and b) and immunofluorescence staining (Fig. 3c and d), myocyte hypertrophy (Fig. 3e and f), as indicated by wheat germ agglutinin plasma membrane staining, and the expression of the fibrosis markers Collagen I, α-SMA, and Periostin (Fig. 3g and h, Fig. S2B), compared to the samples from Ang II-treated mice. Echography was performed to assess LA dimensions. Even though there was no significant difference in ejection fraction between the groups (Fig. S2C and S2D), Ang II infusion led to LA enlargement, and LA volume was decreased following iloprost treatment (Fig. 3i, Fig. S2E). Moreover, immunofluorescent staining for Ki67 confirmed a lower number of proliferating fibroblasts in the Ang II/iloprost-treated atrium compared with that of the Ang II group (Fig. S2F and S2G). These findings provide strong evidence for the potential therapeutic effects of PGI_2_ on AF.

### PGI_2_ attenuated Ang II-induced collagen synthesis and differentiation of atrial fibroblasts

**Fig. 4:**
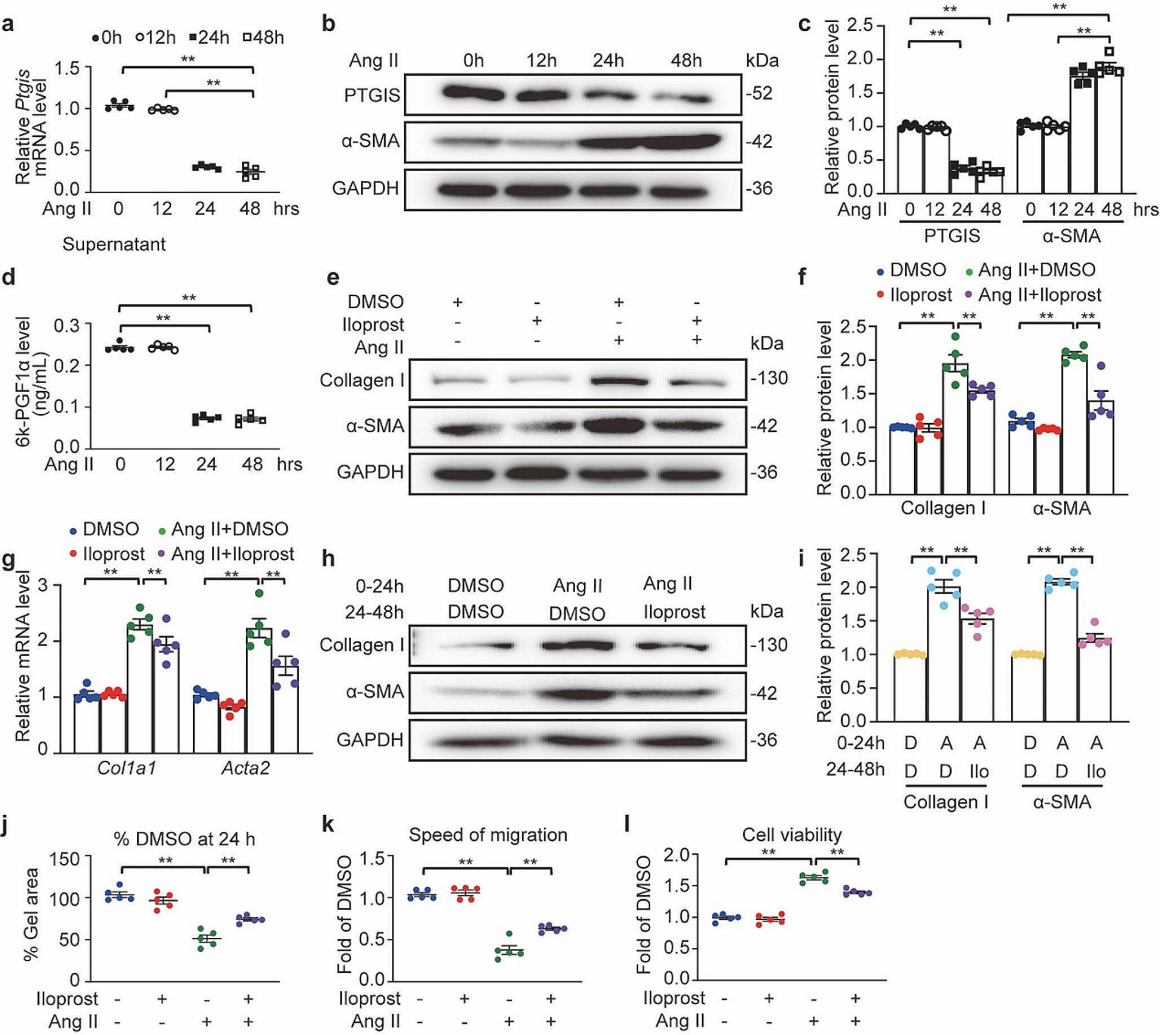
PGI_2_(prostaglandin I_2_) analog iloprost inhibited Ang II (angiotensin II)-induced atrial fibroblast activation. **a** through **d**, Mouse primary atrial fibroblasts were starved for 24 h and then treated with 1 µM Ang II (angiotensin II) for times indicated. One-way ANOVA, *n* = 5, ^**^P < 0.01. **a**, Quantification of *Ptgis* (prostaglandin I synthase) mRNA level. **b** and **c**, Western blot analysis (**b**) and quantification (**c**) of PTGIS and α-SMA (alpha-smooth muscle actin) protein levels. **d**, Liquid chromatography-tandem mass spectrometry detection of supernatant levels of PGI_2_ (determined as its derivative 6k-PGF1α). **e** through **g**, Mouse primary atrial fibroblasts were starved for 24 h and then treated with Ang II (1 µM) and/or iloprost (10 µM) for indicated time pionts. **e** and **f**, Western blot analysis (**e**) and quantification (**f**) of Collagen I and α-SMA protein levels at 24 h. **g**, Quantification of *Col1a1* (collagen type I alpha 1 chain) and *Acta2* (actin alpha 2) mRNA levels at 8 h. **h** and **i**, Mouse primary atrial fibroblasts were starved for 24 h and then treated with Ang II (1 µM) for 24 h, washed and then treated by DMSO or iloprost (10 µM) for another 24 h. Western blot analysis (**h**) and quantification (**i**) of Collagen I and α-SMA protein levels. **j** though **l**, Mouse primary atrial fibroblasts were starved for 24 h and then treated with Ang II (1 µM) and/or iloprost (10 µM) for 24 h. **j**, Cells were embedded in collagen gel lattices before treatment and their contraction was measured after 24 h. Values were expressed as % of DMSO-treated gel area at 24 h. **k**, Migration distances was measured after treatment, value were shown as fold of DMSO-treated distance at 24 h. **l**, Values of cell viability as fold of DMSO-treated cell number at 24 h. Two-way ANOVA, *n* = 5, ^**^P < 0.01

To address how PGI_2_ exerts its antifibrosis role, we first compared PTGIS protein levels in mouse primary atrial cardiomyocytes and fibroblasts and found that PTGIS was highly enriched in the fibroblasts (Fig. S3a and S3b). Then, we saw that the expression of PTGIS decreased at both the mRNA and protein levels in atrial fibroblasts (Fig. 4a to c), as well as PGI_2_ production (Fig. 4d), in response to Ang II, which appears to be a potent stimulator of pro-fibrotic pathways during AF [31]. Given that the proliferation of atrial fibroblasts and their differentiation into myofibroblasts are central to the pathogenesis of atrial remodeling [32], we investigated whether PGI_2_ could inhibit Ang II-induced differentiation of atrial fibroblasts. The Ang II-stimulated increases in the expression of ECM components, collagen type I, and the myofibroblast marker α-SMA, were suppressed by iloprost (Fig. 4e to g). The ability of PGI_2_ to reverse the established fibrotic phenotype in atrial fibroblasts was also investigated. Atrial fibroblasts were first treated with Ang II for 24 h, washed, and subsequently treated with the vehicle or iloprost for an additional 24 h. Fibroblasts treated with Ang II and iloprost expressed less collagen type I and α-SMA than those treated with Ang II and the vehicle (Fig. 4h and i).

### PGI_2_ prevented Ang II-induced contraction, migration, and proliferation of atrial fibroblasts

The ability of fibroblasts to reorganize and contract collagen matrices in vitro is a tool for studying fibroblastic cell behavior [33]. Ang II treatment resulted in a pronounced myofibroblast-mediated contraction of the collagen gel, which was partially prevented by iloprost treatment (Fig. 4j, Fig. S3C). Moreover, co-treatment of atrial fibroblasts with Ang II and iloprost significantly attenuated Ang II-induced fibroblast migration (Fig. 4k, Fig. S3D) and proliferation (Fig. 4l, Fig. S3E).

### PGI_2_ inhibited MAPK pathway on Ang II-induced expression of Il-6 gene in atrial fibroblasts

**Fig. 5:**
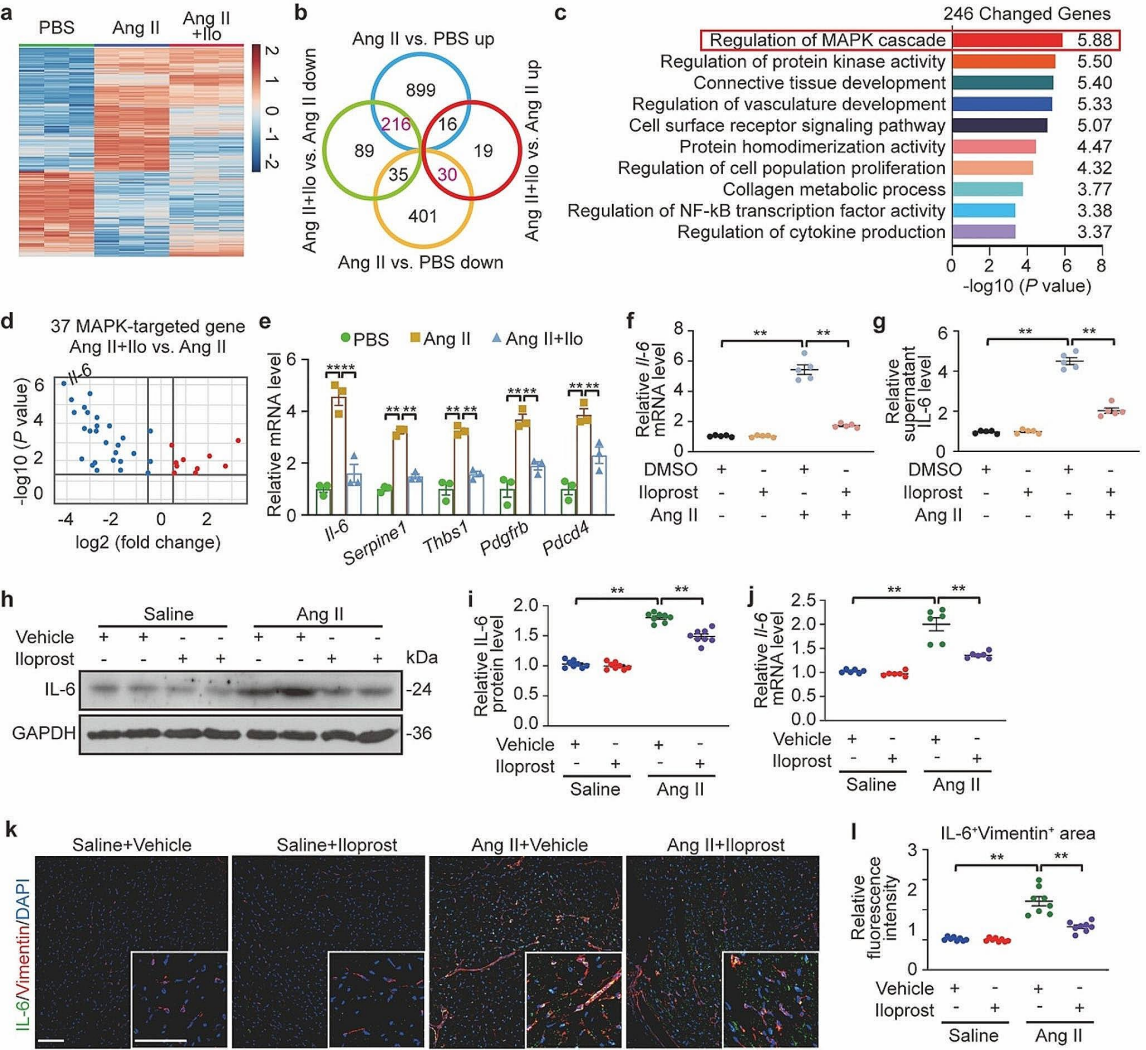
PGI_2_(prostaglandin I_2_) analog iloprost decreased Ang II (angiotensin II)-induced IL-6 (interleukin-6) production in atrial fibroblasts and atrial tissues in mice. **a**, Mouse primary atrial fibroblasts were starved for 24 h and then treated with Ang II (1 µM) and/or iloprost (10 µM) for 8 h. Heatmap depicted expression of genes identified as differentially expressed in the Ang II/DMSO versus PBS/DMSO and their change after treatment with Ang II/Iloprost. **b**, Venn diagram showing differentially regulated genes in response to Ang II with or without iloprost. **c**, Enrichment of potential signaling pathways subjected to Kyoto Encyclopedia of Genes and Genomes pathway analysis of differentially expressed genes from the intersection of Ang II vs. PBS up with Ang II/Iloprost vs. Ang II down (*n* = 216) and Ang II vs. PBS down with Ang II/Iloprost vs. Ang II up (*n* = 30). **d**, Volcano plot of MAPK (mitogen-activated protein kinase) cascade-modulated differentially expressed genes. **e**, Quantification of mRNA levels of the top 5 changed genes in MAPK cascades. Two-way ANOVA, *n* = 3, ^**^P < 0.01. **f** and **g**, Mouse primary atrial fibroblasts were starved for 24 h and then treated with Ang II (angiotensin II, 1 µM) and/or iloprost (10 µM) for either 8 h to measure mRNA levels of *Il-6* (**f**), or 24 h to measure supernatant level of IL-6 (**g**). Two-way ANOVA, *n* = 5, ^**^P < 0.01. **h** and **i**, Western blot analysis (**h**) and quantification (**i**) of IL-6 protein levels in the atrial tissues. Two-way ANOVA, *n* = 8, ^**^P < 0.01. **j**, Quantification of *Il-6* mRNA levels in the atrial tissues. Two-way ANOVA, *n* = 6, ^**^P < 0.01. **k** and **l**, Representative immunofluorescence images (**k**) of atrium sections stained with IL-6 and Vimentin from Saline/Vehicle, Saline/Iloprost, Ang II/Vehicle and Ang II/Iloprost mice (Scale bare, 50 μm) and quantification (**l**) of relative fluorescence intensities of colocalization. Two-way ANOVA, *n* = 8, ^**^P < 0.01

The molecular mechanism underlying the anti-fibrotic effect of PGI_2_ signaling is not well understood. Therefore, RNA-Seq was performed to assess the transcriptional changes in Ang II-treated atrial fibroblasts caused by iloprost treatment. The RNA-seq data showed that a total of 1597 genes were differentially expressed in Ang II-treated cells compared to PBS-treated cells, and 405 genes were differentially expressed in Ang II/iloprost-treated cells compared to Ang II-treated cells (Fig. 5a). To better understand how iloprost changed the transcriptional profile we intercompared these data sets. We found that 216 genes were upregulated in the Ang II-treated cells but were downregulated in the presence of iloprost, and 30 genes were downregulated in the Ang II-treated cells but upregulated after Ang II/iloprost cotreatment (Fig. 5b). Then, these 246 genes were subjected to the Kyoto Encyclopedia of Genes and Genomes pathway enrichment analysis (Fig. 5c), and the differentially regulated genes were significantly enriched for the MAPK pathway. Therefore, MAPK target genes and how they were differentially regulated by Ang II and iloprost were further investigated, and 37 genes were obtained (Fig. 5d). Of these genes, the expression changes of *Il-6*, *Serpine1*, *Thbs1*, *Pdcd4*, and *Pdgfrb* were significantly attenuated by iloprost treatment (Fig. 5e). Notably, IL-6, one of the most common inflammatory cytokines associated with the prothrombotic state of chronic AF [34], was the top differentially regulated gene (Fig. 5d and e). Total cellular *Il-6* mRNA and supernatant IL-6 were markedly increased in Ang II-treated atrial fibroblasts and were attenuated by 70% following co-treatment with Ang II and iloprost (Fig. 5f and g). Moreover, treatment with iloprost after Ang II infusion markedly decreased the gene expression and protein production of IL-6 in atrial tissue (Fig. 5h and l). These findings indicate that the MAPK/IL-6 axis is a crucial downstream target for PGI_2_ anti-fibrotic effects.

### PGI_2_ suppressed Ang II-induced activation ERK1/2 and P38 in a PKA-dependent way

**Fig. 6:**
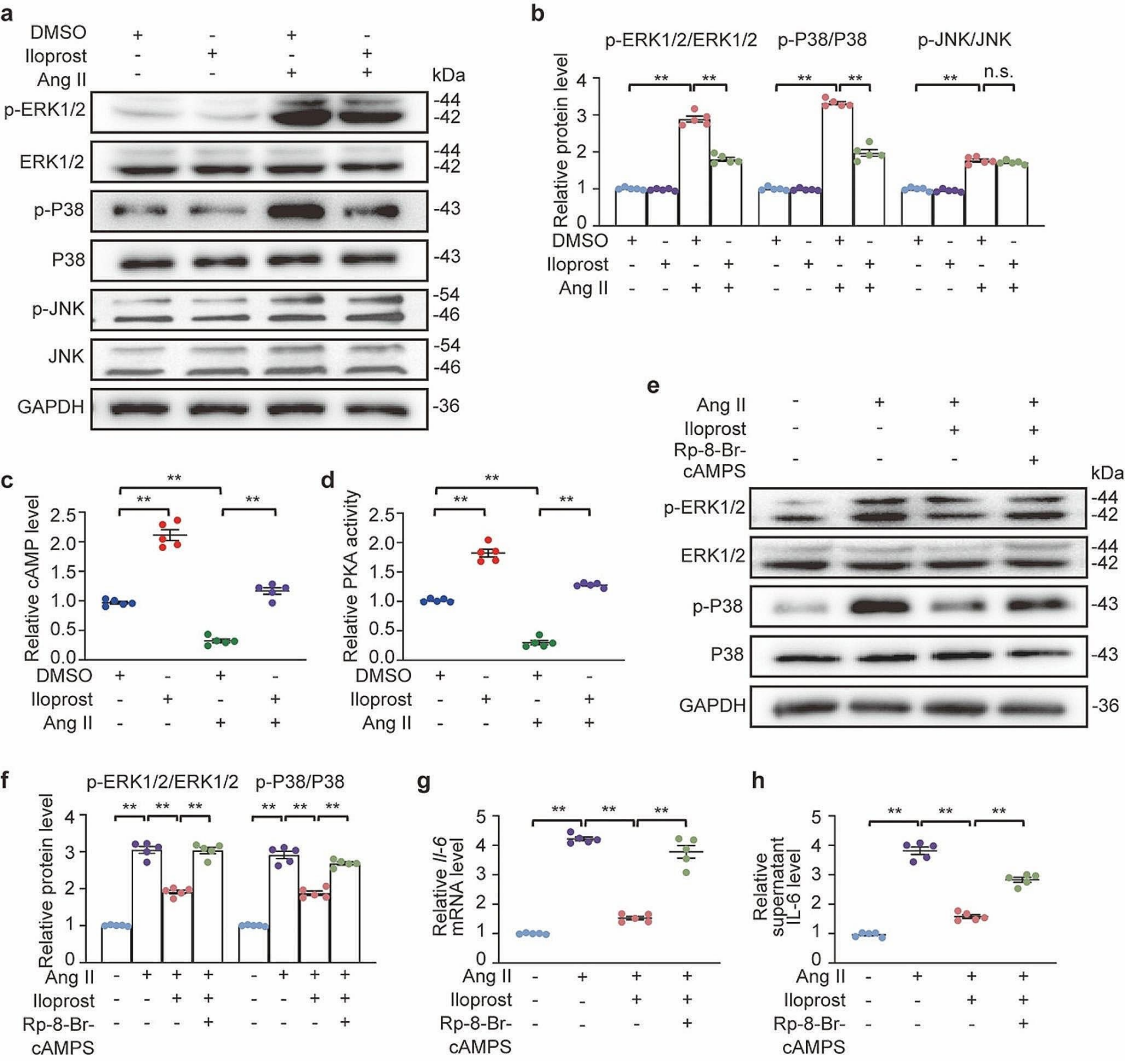
PGI_2_(prostaglandin I_2_) analog iloprost inactivated ERK1/2 (extracellular signal-regulated kinase1/2) and P38 for IL-6 (interleukin-6) production in atrial fibroblasts. **a** through **d**, Mouse primary atrial fibroblasts were starved for 24 h and then treated with Ang II (angiotensin II, 1 µM) and/or iloprost (10 µM) for indicated time points. **a** and **b**, Western blot analysis (**a**) and quantification (**b**) of ERK1/2, P38, and JNK phosphorylation at 30 min. **c**, Quantification of the supernatant level of cAMP (cyclic adenosine monophosphate) at 30 min. **d**, Quantification of PKA (protein kinase A) activity at 30 min. **e** through **h**, Mouse primary atrial fibroblasts were starved for 24 h. Then the PKA inhibitor Rp-8-Br-cAMPS (0.2 mM) was added to cells 2 h before treatment with Ang II (1 µM) and iloprost (10 µM) for indicated time points. **e** and **f**, Western blot analysis (**e**) and quantification (**f**) of ERK1/2 and P38 phosphorylation at 30 min. **g**, Quantification of *Il-6* mRNA level at 8 h. **h**, Quantification of supernatant level of IL-6 at 24 h. Two-way ANOVA, *n* = 5, ^**^P < 0.01

Next, we further explored how PGI_2_ influences the MAPK signaling pathway. A previous study demonstrated that PGI_2_ derivatives exhibit anti-fibrotic effects and showed that these effects were due to cAMP/ PKA-dependent inhibition of the Ras/ERK kinase (MEK)/ERK pathway [35]. Therefore, we first verified whether iloprost had the same effect in atrial fibroblasts. Primary mouse atrial fibroblasts were exposed to Ang II with or without iloprost for 30 min. Ang II-induced phosphorylation of ERK1/2, P38, and JNK was observed, and this phosphorylation was inhibited by iloprost, except for JNK (Fig. 6a and b). Moreover, iloprost incubation led to an increase in intracellular cAMP levels (Fig. 6c) and subsequent activation of PKA (Fig. 6d), indicating the initiation of canonical cAMP-mediated downstream signaling pathways by iloprost. Adenosine-3’,5’-cyclic monophosphorothioate (Rp-cAMPS) is a potent and specific competitive inhibitor of PKA activation by interacting with the cAMP binding site [36]. Atrial fibroblasts were serum-starved overnight and then pretreated with Rp-8Br-cAMPS. Two hours later, Ang II was added, with or without iloprost. The pretreatment with Rp-8Br-cAMPS hindered the ability of iloprost to suppress ERK1/2 and P38 phosphorylation (Fig. 6e and f) as well as IL-6 production (Fig. 6g and h). Hence, iloprost suppresses Ang II-induced IL-6 production through the inhibition of MAPK activation in a PKA-dependent manner.

### Deficiency of IP receptor in atrial fibroblasts promoted fibroblast activation and IL-6 production

**Fig. 7:**
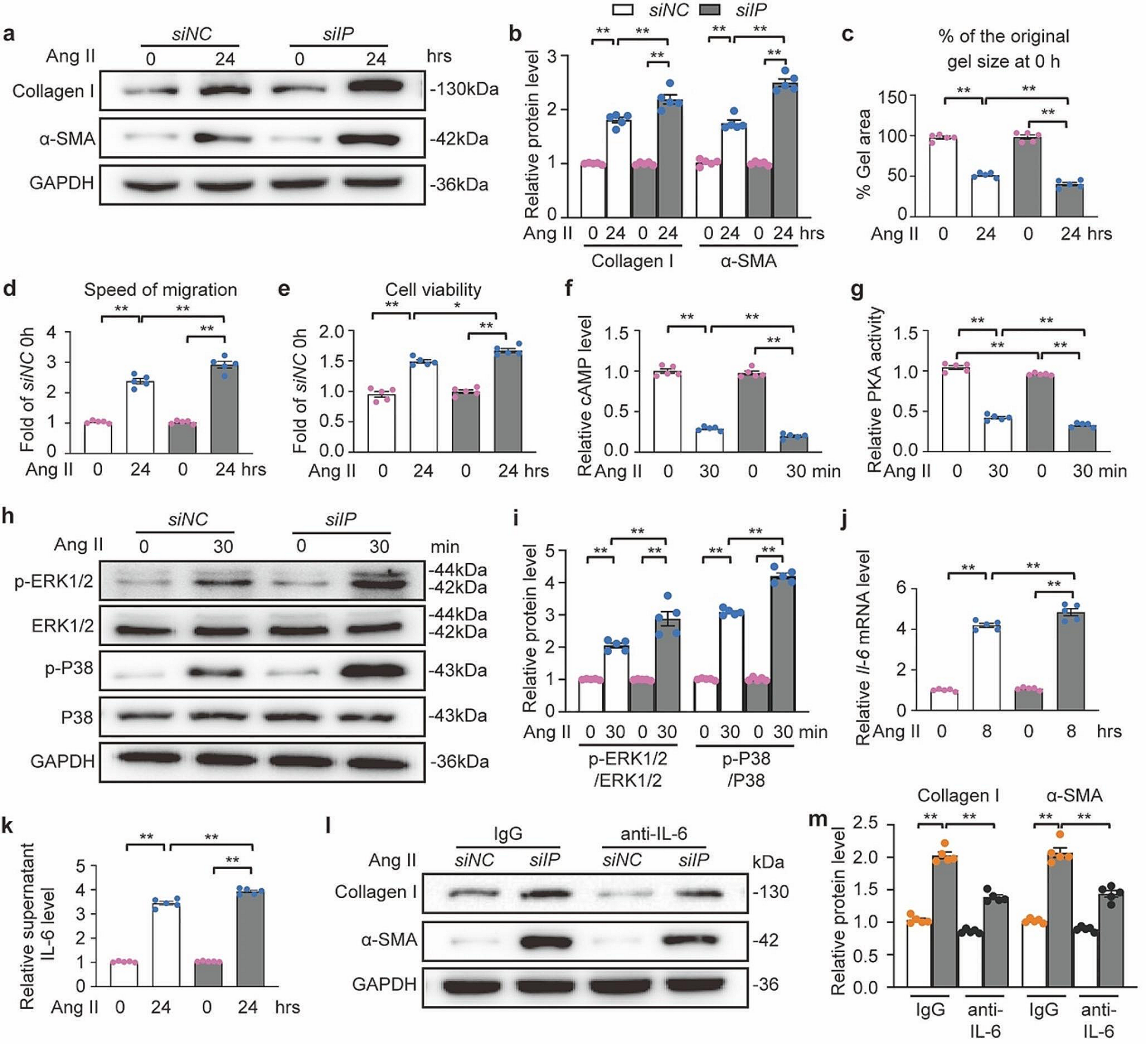
IP (prostaglandin I receptor)-deficient atrial fibroblasts enhanced fibroblasts differentiation and activation of ERK1/2 (extracellular signal-regulated kinase1/2) and P38 for IL-6 (interleukin-6) production. **a** though **k**, Mouse primary atrial fibroblasts were transfected with *siNC* or *siIP* for 48 h and simultaneously treated with Ang II (angiotensin II, 1 µM) for indicated time points. **a** and **b**, Western blot analysis (**a**) and quantification (**b**) of Collagen I and α-SMA (alpha-smooth muscle actin) protein levels. **c**, Values of cell contraction was expressed as % of the original gel area at 0 h. **d**, Values of migration distances was shown as fold of distance at 0 h. **e**, Values of cell proliferation was displayed as fold of cell number at 0 h. **f**, Quantification of supernatant level of cAMP (cyclic adenosine monophosphate). **g**, Quantification of PKA (protein kinase A) activity. **h** and **i**, Western blot analysis (**h**) and quantification (**i**) of ERK1/2 and P38 phosphorylation. **j**, Quantification of *Il-6* mRNA level. **k**, Quantification of supernatant level of IL-6. **l** and **m**, Mouse primary atrial fibroblasts were transfected with *siNC* or *siIP* for 48 h. Then, the cells were pretreated for 30 min with an IL-6 antibody (0.1 µg/mL) or IgG Isotype (0.1 µg/mL) as a control, followed by treated with 1 µM Ang II for 24 h. Western blot analysis (**l**) and quantification (**m**) of Collagen I and α-SMA protein levels. Two-way ANOVA, *n* = 5, ^**^P < 0.01

PGI_2_ selectively binds and activates a G protein-coupled receptor known as the IP receptor to induce its physiological effects. To investigate whether loss of the IP receptor in atrial fibroblasts could induce fibroblast activation, the IP receptor was knocked down in primary mouse atrial fibroblasts via siRNA delivery (Fig. S4A through S4C). IP receptor deficiency increased the expression of collagen type I and α-SMA, indicating the induction of the fibroblast-to-myofibroblast transition (Fig. 7a and b, Fig. S4D and S4E). Moreover, the loss of the IP receptor led to pronounced myofibroblast-mediated contraction of the collagen gel (Fig. 7c, Fig. S4F), increased fibroblast migration in the wound-healing assay (Fig. 7d, Fig. S4G), and proliferation rate (Fig. 7e). We confirmed that IP receptor knockdown resulted in reduced cAMP levels (Fig. 7f) and PKA activity (Fig. 7g), which in turn led to the activation of ERK1/2 and P38 (Fig. 7h and i), subsequently increasing Ang II-induced IL-6 production (Fig. 7j and k) in atrial fibroblasts. Moreover, pretreatment of Ang II-stimulated fibroblasts with anti-IL-6 antibodies suppressed collagen type I and α-SMA expression compared to IgG treatment in atrial fibroblasts with IP receptor knockdown (Fig. 7l and m, Fig. S4H). These results provide evidence that loss of the IP receptor activates the MAPK pathway and contributes to the production of IL-6, leading to atrial fibroblast activation.

### Knockdown of the IP receptor in cardiac fibroblasts aggravated Ang II-induced AF and atrial fibrosis

**Fig. 8:**
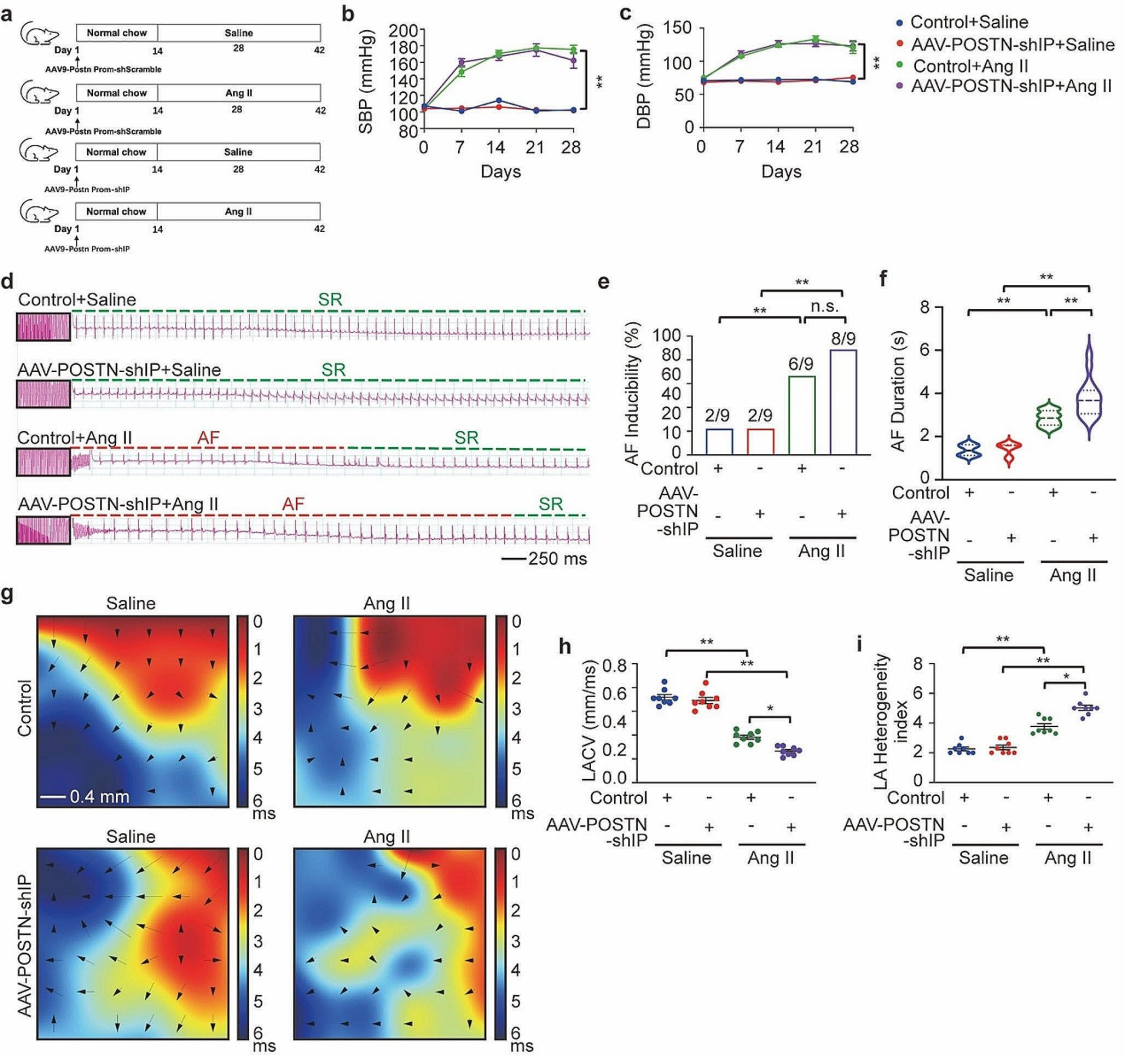
Cardiac fibroblast-specific IP (prostaglandin I receptor) knockdown aggravated Ang II (angiotensin II) -induced AF (atrial fibrillation) inducibility. **a**, C57BL/6J mice were administered with adeno-associated virus serotype 9 (AAV9) carrying periostin promoter-derived IP shRNA, allowing the AAV9 vector to selectively direct IP expression specifically to cardiac fibroblasts, termed AAV-POSTN-shIP. Mice assigned to the control group received injections of rAAV9 containing scrambled shRNA. Following viral infection, all mice underwent a 28-day infusion period with either Ang II (at a dose of 2000 ng/kg per min) or saline solution. **b** and **c**, Noninvasive tailed-cuff monitoring of SBP (systolic blood pressure, **b**) and DBP (diastolic blood pressure, **c**). **d**, Representative surface ECG (electrocardiogram) tracing during burst pacing (BP) is depicted within the black box. Red dashes denote AF, and green dashes denote sinus rhythm (SR). **e**, Fraction of animals with successful AF induction. Ratios represent the number of AF induced to total animals. *n* = 9 per group, Fisher exact test. **f**, Violin plot of AF in animals (numbers for each group are shown in e). **g**, Representative epicardial electrical conduction activation maps of the left atrium. The color bar on the right side of the image represents the conduction proceeding advancing from 0 ms (dark red) to the end of imaging period (6 ms, blue). The arrows indicate the direction of conduction. Scale bare, 0.4 mm. **h**, Summary of CV (conduction velocity) in the left atrium. **i**, Summary of inhomogeneity index in the left atrium. Two-way ANOVA, *n* = 8–10 mice per group, ^*^P < 0.05, ^**^P < 0.01

**Fig. 9:**
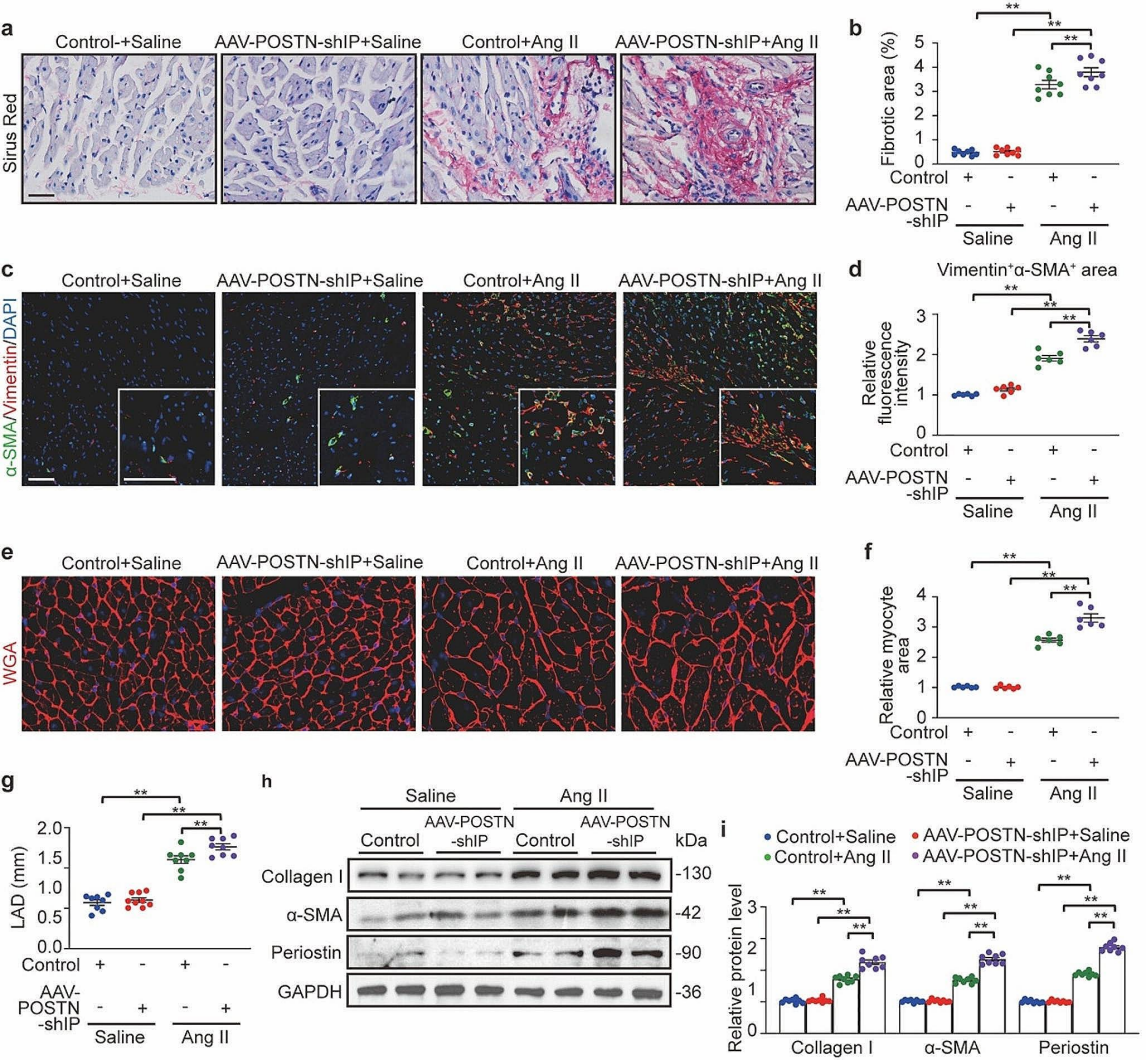
Cardiac fibroblast-specific IP (prostaglandin I receptor) knockdown aggravated Ang II (angiotensin II) -induced atrial fibrosis. **a**, Representative Sirus red staining of left atrial tissues. Scale bare, 50 μm. **b**, Quantification of the fibrotic area. **c**, Representative immunofluorescence staining of α-SMA (alpha-smooth muscle actin) and Vimentin. in left atrial tissues. Scale bare, 50 μm. **d**, Quantification of relative fluorescence intensities of colocalization. **e**, Alexa Fluor 594-conjugated wheat germ agglutinin (WGA) staining of left atrial tissues. Scale bare, 50 μm. **f**, Quantification of the myocyte area. **g**, Quantification of LAD (left atrium diameter). **h** and **i**, Western blot analysis (**h**) and quantification (**i**) of Collagen I, α-SMA and Periostin protein levels in the atrial tissues. Two-way ANOVA, *n* = 6–8 mice per group, ^*^P < 0.05, ^**^P < 0.01

Previous in vivo studies have demonstrated that mice lacking IP receptors show increased cardiomyocyte hypertrophy and cardiac fibrosis in response to pressure overload [15], elevated blood pressure, impaired cardiac function, and increased cardiac fibrosis during high-salt feeding [37], and increased myocardial infarct size after ischemia-reperfusion injury [38]. However, the effect of IP receptor absence on atrial fibrosis and AF in the presence of Ang II has not been explored. Here, we established a mouse model with knockdown of IP in cardiac fibroblasts by injecting C57BL/6J mice with AAV9 carrying a periostin promoter-driven IP shRNA, designated as AAV-POSTN-shIP. This approach ensured selective targeting of IP expression specifically to cardiac fibroblasts. As a Control, mice were injected with AAV9 containing scrambled shRNA. Following the viral vector injections, all animals were then administered either Ang II or saline for 28 days (Fig. 8a). The AAV-POSTN-shIP mice demonstrated an 80% decrease in IP receptor expression in atrial tissue compared Control mice (Fig. S5A and S5B). The elevation of SBP and DBP was similar between Ang II-treated AAV-POSTN-shIP and Control mice (Fig. 8b and c). After Ang II infusion, AF inducibility (Fig. 8d) and duration (Fig. 8f) were increased in AAV9-POSTN-shIP mice compared to Control mice (Control + Ang II: 2.850 ± 0.100 s versus AAV9-POSTN-shIP + Ang II: 3.775 ± 0.211 s). However, there was no significant difference in AF inducibility between the two groups (6/9 for Control + Ang II versus 8/9 for AAV-POSTN-shIP + Ang II) (Fig. 8e). LA conduction velocity and dispersion were significantly more disordered in AAV-POSTN-shIP mice than in Control mice after Ang II infusion (Fig. 8g and i). Additionally, a comparison was conducted between Ang II alone and Ang II combined with iloprost in the absence of IP receptors regarding AF inducibility and AF duration. Each group consisted of 9 mice, with 8 mice in each group showing induced AF. No statistically significant difference was observed in the duration of AF between the two groups (Ang II alone in the absence of IP receptors: 3.779 ± 0.31 s versus Ang II with iloprost in the absense of IP receptors:3.535 ± 0.23 s). This suggests that PGI_2_ exerts its anti-AF effect mainly through IP receptor activation. Moreover, the atrial fibrotic area (Fig. 9a and d) and myocyte area (Fig. 9e and f), LA diameters (Fig. 9g and Fig. S5C), and expression of Collagen I, α-SMA, and Periostin in atrial tissue were increased in Ang II-treated AAV-POSTN-shIP mice compared with Control mice (Fig. 9h and i, Fig. S5D), but there was no significant difference in ejection fraction (Fig. S5E and S5F).

**Fig. 10:**
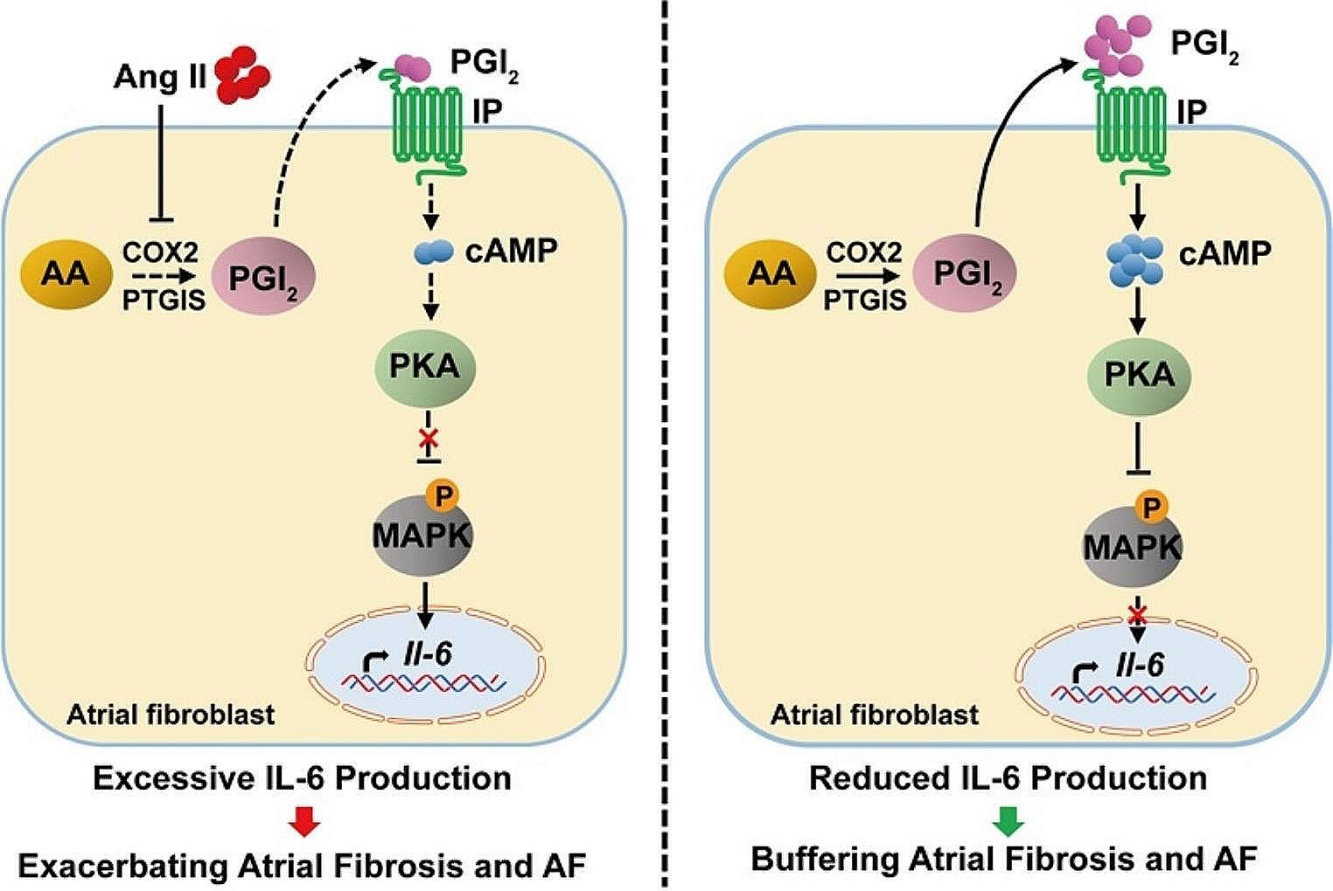
Working model for the anti-fibrotic role of PGI_2_(prostaglandin I_2_) in Ang II (Angiotensin II)-induced AF (atrial fibrillation) and atrial fibrosis. PGI_2_ alleviates atrial fibrosis and development of AF by elevating cAMP levels to enhance PKA (protein kinase A) activity, thus suppressing the activation of ERK1/2 (extracellular signal-regulated kinase1/2) and P38, leading to inhibition of IL-6 (interleukin-6) production. MAPK (mitogen-activated protein kinase)

## Discussion

PGI_2_ is found widely in the body, and has been studied in pulmonary and cardiac vasculature, peripheral vasculature, and various immune cells. As an endogenous vasodilator and a potent inhibitor of platelet aggregation, reduced levels of PGI_2_ have been reported to be a risk factor for high blood pressure, and myocardial and cerebral infarction [39]. In the present study, we identified the eicosanoid profile alternations by comparing the metabolism in patients with AF and corresponding healthy controls. Overall, 9 distinct eicosanoids were deregulated in patients with AF. PGI_2_ content was drastically reduced in patients’ plasma, which is generated from PGH_2_ by the action of the enzyme PTGIS. PTGIS is a member of the cytochrome P450 enzyme family and a membrane protein that localizes to the endoplasmic reticulum. It is widely expressed in various tissues. Pan et al. have reported that PTGIS overexpression inhibited transforming growth factor-β1 (TGF-β1)-induced activation of hepatic stellate cells and alleviated carbon tetrachloride-induced liver fibrosis in C57BL/6J mice [40]. We demonstrate that atrial fibroblast PTGIS expression was downregulated in the atrial tissue of patients with PAF and the animal model featuring atrial fibrosis, indicating that atrial fibroblast may be an important source of PGI_2_ production. Low levels of PTGIS and, in turn, reduced PGI_2_ content may contribute to a higher risk of incident AF and atrial structural abnormalities. Identification of biomarkers associated with AF may improve our understanding of the key pathophysiological mechanisms of arrhythmia and facilitate the identification of potential therapeutic targets. Recently, biomarkers such as N-terminal pro-B-type natriuretic peptide, C-reactive protein, fibroblast growth factor-23, and high-sensitivity troponin I have been shown to be associated with AF and found to improve risk prediction [41]. It is conceivable that 6k-PGF1α can serve as a biomarker of AF.

PGI_2_ binds to IP, a G-protein-coupled receptor that is found primarily on the cell membranes of platelets, smooth muscle cells, and some immune cells. Receptor binding and G-protein activation trigger an increase in intracellular cAMP, which activates PKA, to perform its function. Evidence indicates that cAMP-regulated signaling pathways profoundly affect cardiac fibroblast function and impact the development of cardiac fibrosis [16, 42]. Prior work shows that cAMP signaling inhibits LA fibroblast collagen production and may be a novel target for pro-fibrillatory cardiac remodeling [43]. In cultured cardiac fibroblasts, treatment with PGI_2_ analog beraprost and cicaprost induce a cAMP elevating effect, such as inhibiting collagen synthesis, but have opposing actions on fibroblast differentiation [16]. Consistent with these findings, iloprost, another synthetic analog of PGI_2_, prevented atrial fibroblasts-to-myofibroblasts differentiation and produced antifibrotic effects, including collagen synthesis and attenuation proliferation via increasing intercellular cAMP levels.

Intracellular MAPK signaling cascades play an important role in the pathogenesis of cardiac and vascular disease. The classic MAPK cascades include the ERK1/2 pathway, the P38 MAPK pathway, the JNK pathway, and the ERK5 pathway. In rat and mouse cardiac fibroblasts, inhibition of P38 attenuated TGF-β-induced expression of collagen I and α-SMA and reduced progressive fibrotic burden to help maintain cardiac ventricular performance [44–46]. In Marfan syndrome mice, nonmyocyte ERK signaling promoted load-induced cardiac fibrosis [47]. Stratton et al. found that iloprost reversed the ability of TGF-β2 to induce collagen synthesis in the wound chamber model by suppressing the Ras/MEK/ERK cascade in a PKA-dependent manner [35]. In primary mouse atrial fibroblasts, we used RNA-seq analysis to identify MAPK cascades as a highly regulated pathway by iloprost in response to Ang II, with significant effects on related target gene expression, such as *Il-6*. We reasoned that iloprost inhibited the ERK1/2 and P38 signaling by inducing cAMP elevation and PKA activity.

IL-6, as one of the most important pro-inflammatory cytokines, is closely related to cardiovascular diseases such as atherosclerosis, hypertension, cardiomyopathy, and cardiac fibrosis [48]. In the cardiovascular system, IL-6 is secreted by macrophages, monocytes, endothelial cells, and fibroblasts. IL-6 infusions in vivo resulted in a marked increase in ventricular stiffness and collagen volume fraction (36). In cardiac fibroblasts, IL-6 trans-signaling enhanced fibrosis-related factor expression and also played a role in mediating a phenotypic conversion to myofibroblast [49, 50]. In AF, higher concentrations of IL-6 have been associated with higher AF burden and increased mortality [34, 51–53], suggesting that IL-6 is an important mediator in the pathophysiology of AF. Several studies have demonstrated that Ang II binding to Ang II receptor type 1 stimulates MAPK, thus regulating the transcription of the pro-fibrotic genes *Serpine1*, *Tgfb*, and *Il-6*. This stimulates the proliferation and activation of fibroblasts, leading to increased atrial dilatation and fibrillation [53–55]. Iloprost prevents pulmonary fibrosis, possibly by upregulating anti-fibrotic mediators (interferon γ and C-X-C motif chemokine ligand 10) and downregulating pro-inflammatory and pro-fibrotic cytokines (tumor necrosis factor α, IL-6, and TGF-β1) [56, 57]. In line with these studies, we found that iloprost inhibited the MAPK pathway, including ERK1/2 and P38, and downstream *Il-6* gene transcription in atrial fibroblasts in a cAMP/PKA-dependent mechanism.

Evidence suggests that iloprost, being more stable than PGI_2_, has the same biologic profile as the natural substance concerning prostaglandin receptor binding and cellular effects [58]. Moreover, like PGI_2_, iloprost has poor selectivity for prostanoid receptors, being equipotent at activating both IP and EP1 receptors [59]. Inhaled iloprost improved right ventricular function and reversed established fibrosis by preventing collagen synthesis and increasing collagen turnover in pulmonary artery-banded rats [60]. Iloprost also inhibits pulmonary fibrosis induced by bleomycin and decreases fibrotic changes with greater efficacy than methylprednisolone [61]. ONO-1301, a slow-releasing form of the PGI_2_ analog, has been shown to attenuate pressure-overloaded cardiac fibrosis by inhibiting TGF-β-induced cardiac fibroblast-to-myofibroblast transition via the activation of the IP receptor [62]. These studies imply that PGI_2_ may prevent fibrotic progression, making it suitable for treating fibrotic-related diseases. We noted that enhancement of PGI_2_/IP signaling via a sustained supply of PGI_2_ analog iloprost suppressed Ang II-induced atrial fibrogenesis and AF incidence in mice.

Our work also demonstrated that blockage of PGI_2_/IP signaling by knocking down cardiac IP receptors could exacerbate AF-related phenotypes. These findings may provide causal mechanistic explanations about non-steroidal anti-inflammatory drugs (NSAIDs)-induced AF. NSAIDs are effective in the treatment of heart attacks, ischemic strokes, and blood clots in people at high risk. However, NSAIDs have been associated with an increased risk of AF [63, 64]. The biological explanation is not fully understood but is speculated to involve adverse effects on fluid retention, serum electrolytes, and blood pressure [63]. Since NSAIDs work by inhibiting the activity of COX enzymes, non-selective and COX-2 selective, reduced production of the anti-atherogenic PGI_2_ may contribute to cardiovascular side effects [65]. For example, aspirin has a dose-related effect on PGI_2_ synthesis, with substantial inhibition becoming apparent at doses over 80 mg/day [66, 67]. Therefore, it is indicated that the PGI_2_ analog in combination with NSAIDs can help further reduce the risk of AF.

## Conclusion

The present study demonstrated that the PGI_2_, as a critical endogenous anti-fibrotic regulator, ameliorates Ang II-induced atrial fibrosis and AF by directly arresting atrial fibroblast activation and myofibroblast transdifferentiation, which were aggravated in cardiac fibroblast-specific IP receptor-deficient mice. Mechanistically, PGI_2_ promoted cAMP/PKA axis activation leading to impaired MAPK cascade activity and ultimately causing reduced expression of MAPK target genes and the formation of AF (Fig. 10). Therefore, these findings suggest that PGI_2_ may play a protective role in atrial remodeling, highlighting the PGI_2_/IP receptor system as a potential upstream therapeutic target for treating atrial fibrosis and AF.

### Electronic supplementary material

Below is the link to the electronic supplementary material.

Supplementary Material 1

Supplementary Material 2

## Data Availability

Supplementary figures and tables were listed in Supplemental material file. Metabolomics data, statistical data of patients and RNA-seq data will be made available upon reasonable request.
