# Supplementary material for: Prostaglandin I2 signaling prevents angiotensin II-induced atrial remodeling and vulnerability to atrial fibrillation in mice
Source: Cell Mol Life Sci. 2024 Jun 15;81(1):264. doi: 10.1007/s00018-024-05259-3 (PMC11335301; doi:10.1007/s00018-024-05259-3)
Supplement: Supplementary file 1 — Supplementary Material 1 [file 18_2024_5259_MOESM1_ESM.docx]

**Supplemental material**

**
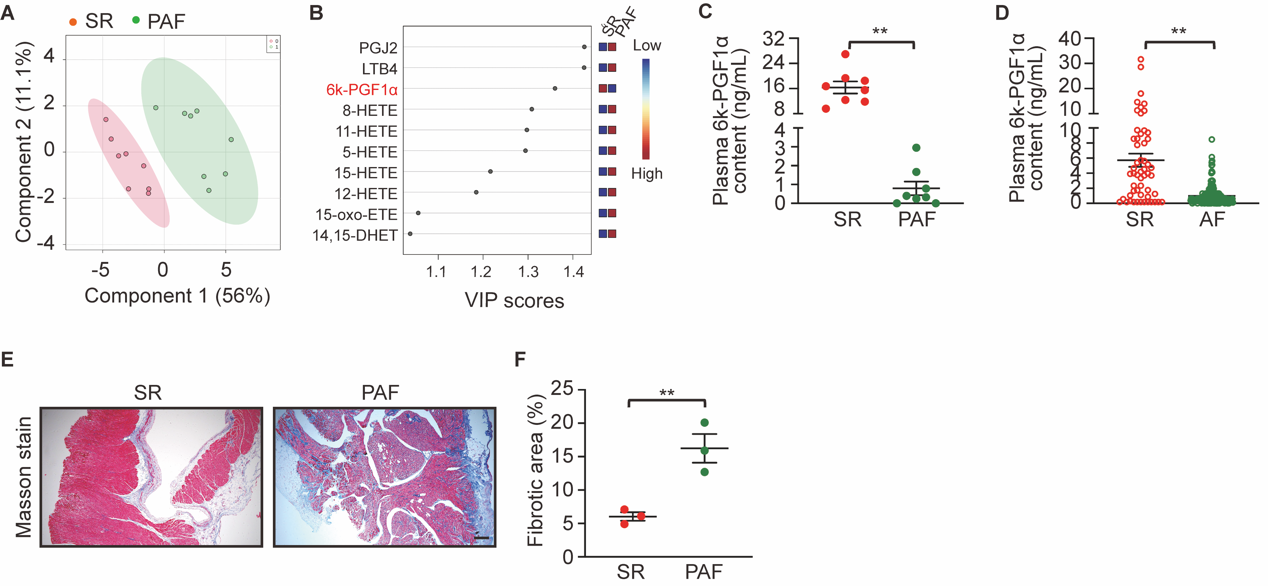
**

**Figure S1. A**, PLS-DA (partial least squares discriminant analysis) has shown an arachidonic acid metabolic profile in the plasma of patients with PAF (persistent atrial fibrillation) and those with normal SR (sinus rhythm). The red plot represents SR group, and the green plot represents PAF group. The samples of these 2 groups were completely separated. **B**, Features (variables) of top 10 most significant metabolites based on VIP (variable important for prediction) scores from PLS-DA. The x-axis shows the correlation scores, and the y-axis corresponds to the metabolites. Color bars show median intensity of variable in the respective group. **C**, LC-MS/MS (liquid chromatography-tandem mass spectrometry) detection of plasma levels of 6k-PGF1α in SR (n=8) and PAF (n=8) groups. Manne-Whitney U test, ^**^*P*<0.01. **D**, LC-MS/MS detection of plasma levels of 6k-PGF1α in SR (n=55) and AF (n=182) groups. Manne-Whitney U test, ^**^*P*<0.01. **E**, Representative Masson trichrome staining (Scale bare, 100 μm) of left atrial tissues from SR control (n=3) and patients with PAF (n=3). **F**, Quantification of the fibrotic area. Unpaired 2-tailed *t*-test, ^**^*P*<0.01.


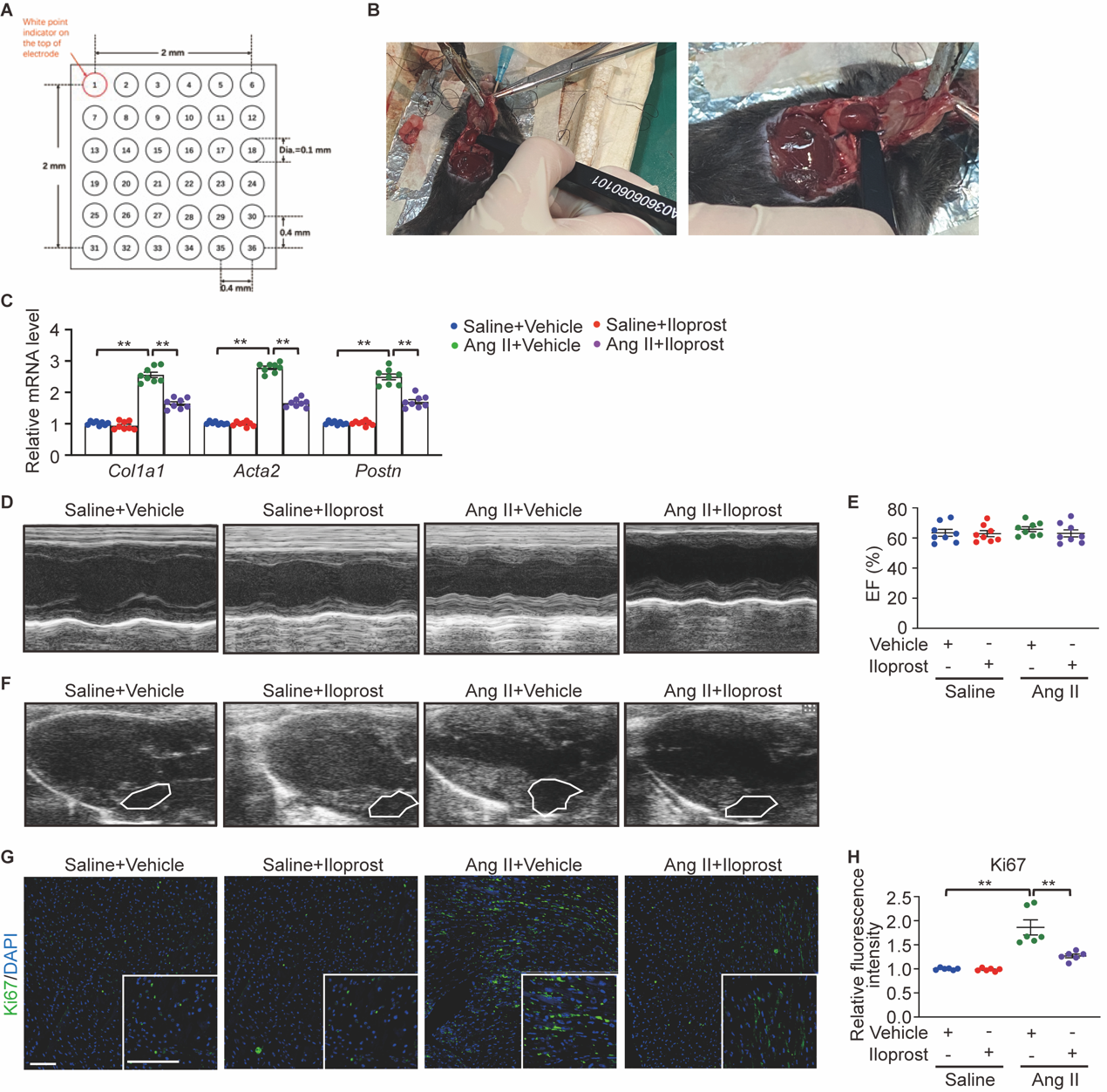


**Figure S2. A**, Multiple-electrode probe array (MEPAs) configuration consisting of 36 electrodes in a square 6×6 matrix, with an electrode diameter 0.1 mm, and interelectrode distance 0.4 mm. **B**, Photograph of MEAPs and the array in situ. **C** through **H,** C57BL/6J mice were housed with or without infusion of Ang II (2000 ng/kg/min) for 28 days. And mice were treated with iloprost (0.2 mg/kg/day) or vehicle from the 15th day after exposure to Ang II for 14 days. **B**, Quantification of *Col1a1* (collagen type I alpha 1 chain), *Acta2* (actin alpha 2) and *Postn* (periostin) mRNA levels in the atrial tissues. **C** and **D**, Representative gross images (**C**) of M-mode echocardiography and quantification (**D**) of EF% (ejection fraction %) in the indicated groups. **E**, Representative gross images of B-mode echocardiography recording of the left atrium. **E** and **F**, Representative immunofluorescence staining (**F**) of Ki67in atrial tissues and quantification (**G**) of relative fluorescence intensities. Scale bare, 50 μm. Two-way ANOVA, n=6–8 mice per group, ^**^*P*<0.01.


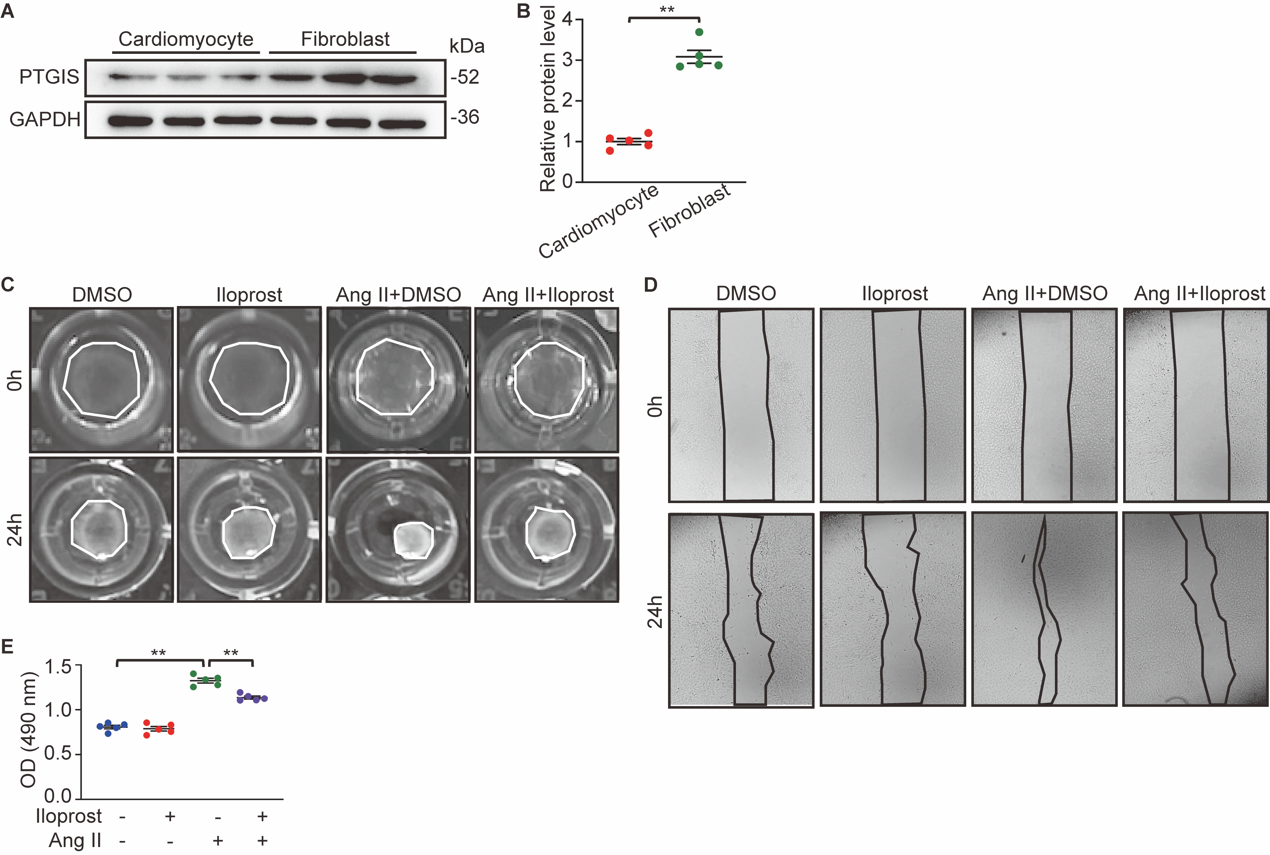


**Figure S3. A** and **B**, Western blot analysis (**A**) and quantification (**B**) of PTGIS protein levels in mouse atrial cardiomyocytes and fibroblasts. Unpaired 2-tailed *t*-test, ^**^*P*<0.01. **C** through **E**, Mouse primary atrial fibroblasts were starved for 24 h and then treated with Ang II (1 μM) and/or iloprost (10 μM) for 24 h. **C**, Cells were embedded in collagen gel lattices and their contraction in response to indicated treatment was measured after 24 h. Representative images of collagen gel contraction were captured at 0 and 24 h. **D**, Representative images of wound-healing migration at 0 and 24 h. **E**, The mean OD (optical density) of the MTS assay was used to calculate the percentage of cell viability. Two-way ANOVA, n=5, ^**^*P*<0.01.


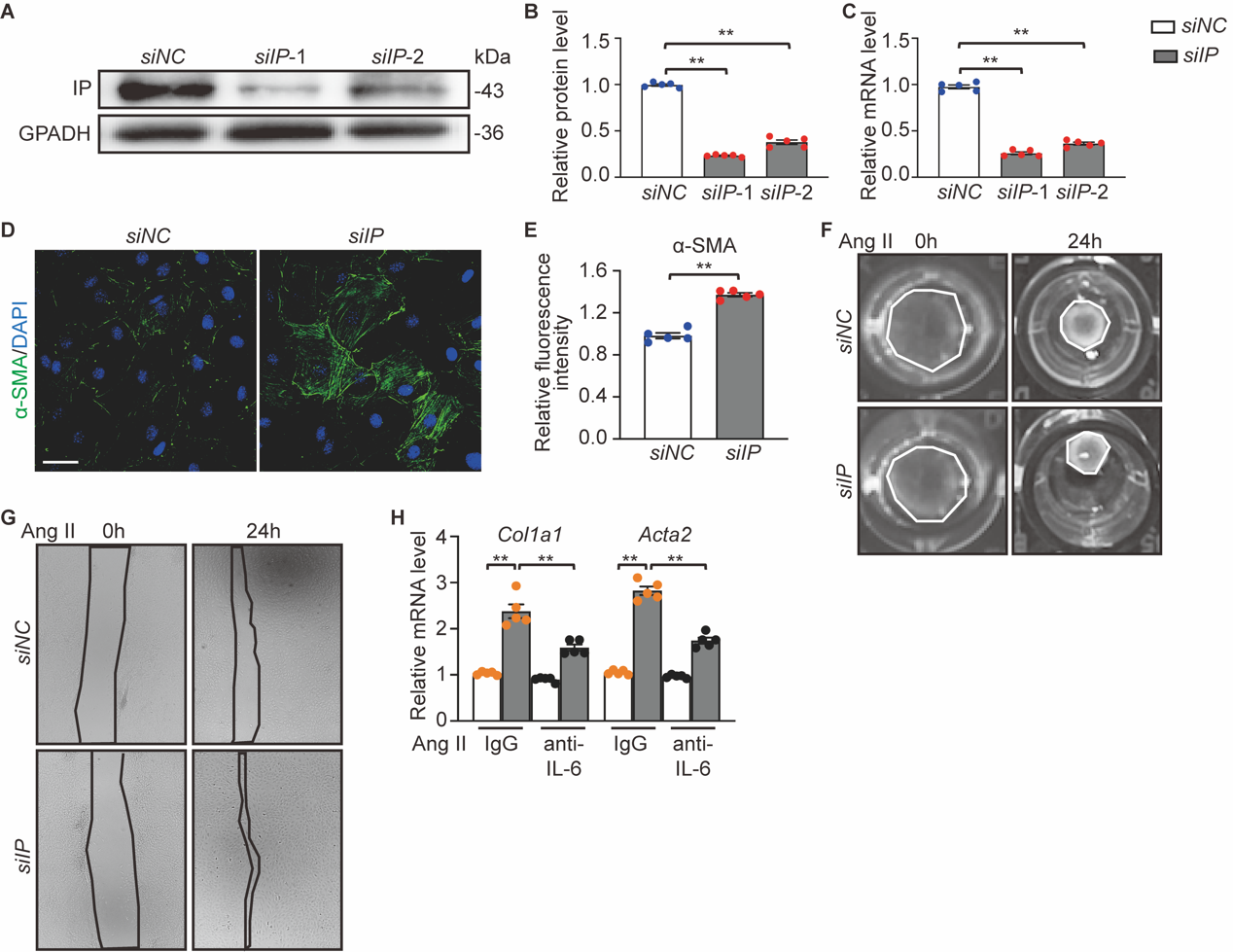


**Figure S4. A** though **C,** Mouse primary atrial fibroblasts were transfected with *siNC* or *siIP* for 48 h. One-way ANOVA, n=5, ^**^*P*<0.01. **A** and **B**, Western blot analysis (**A**) and quantification (**B**) of IP protein levels. **C**, Quantification of *IP* mRNA levels. **D** and **E**, Immunofluorescence staining (**D**) of $\alpha$-SMA (alpha-smooth muscle actin) and quantification (**E**) of relative fluorescence intensities of $\alpha$-SMA. Scale bare, 40 μm. **F** and **G**, Mouse primary atrial fibroblasts were transfected with *siNC* or *siIP* for 48 h and simultaneously treated with Ang II (angiotensin II, 1 μM) for 24 h. **F**, Cells were embedded in collagen gel lattices, representative images of collagen gel contraction were captured at 0 and 24 h. **G**, Representative images of wound-healing migration at 0 and 24 h after Ang II treatment. **H**, Mouse primary atrial fibroblasts were transfected with *siNC* or *siIP* for 48 h. Then, the cells were pretreated for 30 min with an IL-6 antibody (0.1 μg/mL) or IgG Isotype (0.1 μg/mL) as a control, followed by treated with 1 μM Ang II for 8 h. Quantification of *Col1a1* (collagen type I alpha 1 chain) and *Acta2* (actin alpha 2) mRNA levels. Two-way ANOVA, n=5, ^**^*P*<0.01.


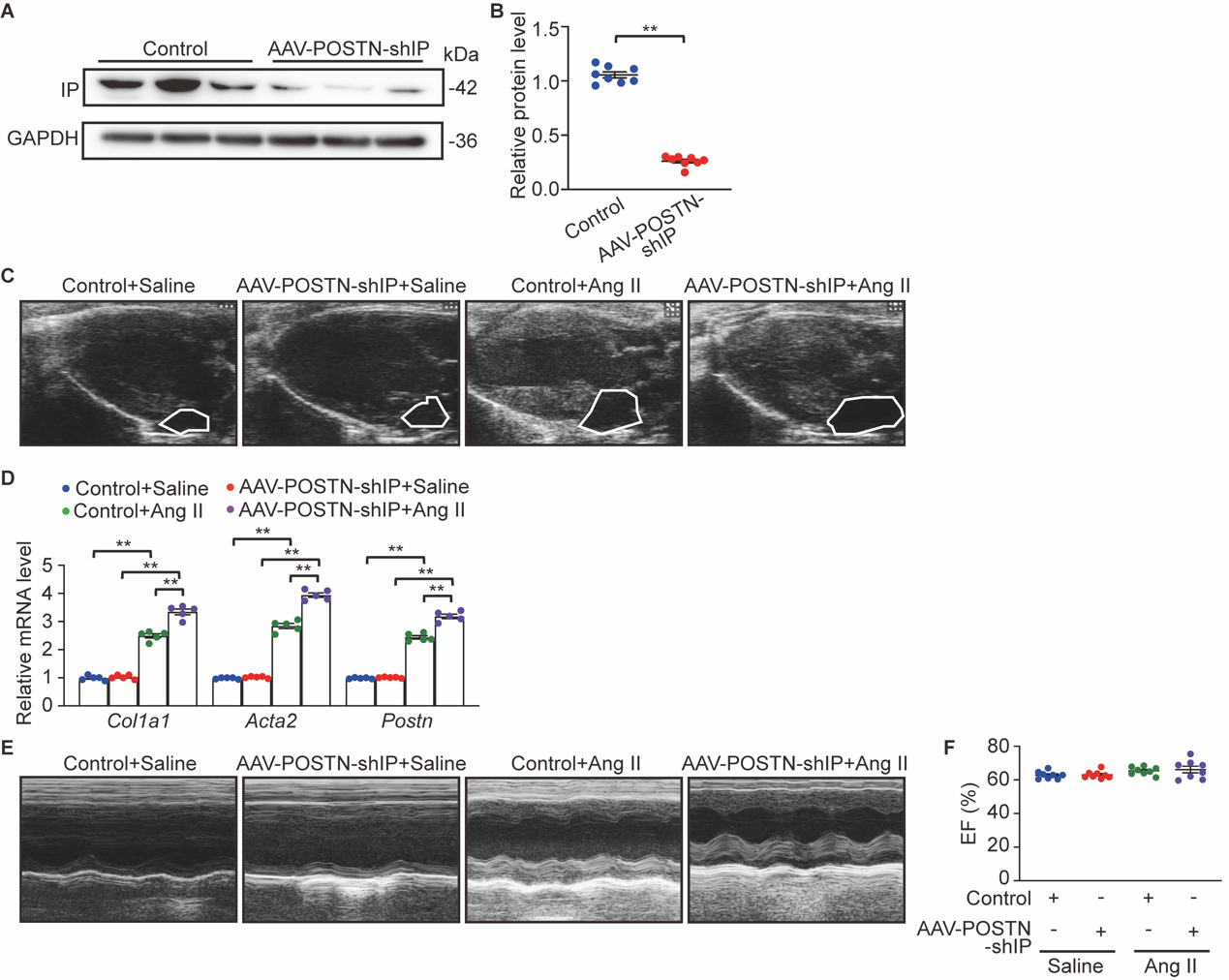


**Figure S5.** C57BL/6J mice, either infected with adeno-associated virus serotype 9 (AAV9) carrying a periostin promoter-driven shRNA targeting IP (AAV9-POSTIN-shIP) or harboring a periostin promoter-scramble construct (control), underwent infusion with either Ang II (2000 ng/kg per min) or saline for 28 days. **A** and **B**, Western blot analysis (**A**) and quantification (**B**) of IP protein levels in the heart tissues at 14 days post rAAV9s injection. Unpaired 2-tail *t*-test, n=8, ^**^*P*<0.01. **C**, Representative gross images of B-mode echocardiography recording of the left atrium. **D**, Quantification of *Col1a1* (collagen type I alpha 1 chain), *Acta2* (actin alpha 2) and *Postn* (periostin) mRNA levels in the atrial tissues. **E** and **F**, Representative gross images of M-mode echocardiography (**E**) and quantification (**F**) of EF% (ejection fraction %) in the indicated groups. Two-way ANOVA, n=8, ^**^*P*<0.01.

**Table S1. Primer sequences**

| Primer | Sense | Antisense |
| --- | --- | --- |
| human *Actb* | CACAGAGCCTCGCCTTTGC | ATATCATCATCCATGGTGAGCTGG |
| mouse *Actb* | GAGGTATCCTGACCCTGAAGTA | CACACGCAGCTCATTGTAGA |
| human *Acta2* | AAAGCAAGTCCTCCAGCGTT | GCTTCACAGGATTCCCGTCT |
| mouse *Acta2* | TCAGGGAGTAATGGTTGGAATG | GGTGATGATGCCGTGTTCTA |
| mouse *Col1a1* | GCTTGAAGACCTATGTGGGTATAA | GGTGGAGAAAGGAGCAGAAA |
| mouse *Il6* | CTTCCATCCAGTTGCCTTCT | CTCCGACTTGTGAAGTGGTATAG |
| mouse *IP* | TGCTCCTGCTGATGTTTCTC | TCTGGAGAGGCTTTGCTAATATG |
| *mouse Postn* | TGTGTATCGGACGGCTATCT | CTCTGCTGGTTGGATGATTTCT |
| human *Ptgis* | GAGAGTTACCTGCTGCACCT | GACCCATATTCCCCTGTGTGG |
| mouse *Ptgis* | CCAGAAGGACCCAGAAATCTAC | TTCAGCCGTTTCCCATCTT |
